# Using Genetic Risk Score Approaches to Infer Whether an Environmental Factor Attenuates or Exacerbates the Adverse Influence of a Candidate Gene

**DOI:** 10.3389/fgene.2020.00331

**Published:** 2020-05-08

**Authors:** Wan-Yu Lin, Yu-Shun Lin, Chang-Chuan Chan, Yu-Li Liu, Shih-Jen Tsai, Po-Hsiu Kuo

**Affiliations:** ^1^Institute of Epidemiology and Preventive Medicine, College of Public Health, National Taiwan University, Taipei, Taiwan; ^2^Department of Public Health, College of Public Health, National Taiwan University, Taipei, Taiwan; ^3^Institute of Environmental and Occupational Health Sciences, College of Public Health, National Taiwan University, Taipei, Taiwan; ^4^Center for Neuropsychiatric Research, National Health Research Institutes, Miaoli, Taiwan; ^5^Department of Psychiatry, Taipei Veterans General Hospital, Taipei, Taiwan; ^6^Division of Psychiatry, National Yang-Ming University, Taipei, Taiwan; ^7^Institute of Brain Science, National Yang-Ming University, Taipei, Taiwan

**Keywords:** body mass index, elastic net regression, gene-environment interaction, lasso, ridge regression

## Abstract

Some candidate genes have been robustly reported to be associated with complex traits, such as the *fat mass and obesity-associated* (*FTO*) gene on body mass index (BMI), and the *fibroblast growth factor 5* (*FGF5*) gene on blood pressure levels. It is of interest to know whether an environmental factor (E) can attenuate or exacerbate the adverse influence of a candidate gene. To this end, we here evaluate the performance of “genetic risk score” (GRS) approaches to detect “gene-environment interactions” (G × E). In the first stage, a GRS is calculated according to the genotypes of variants in a candidate gene. In the second stage, we test whether E can significantly modify this GRS effect. This two-stage procedure can not only provide a *p*-value for a G × E test but also guide inferences on how E modifies the adverse effect of a gene. With systematic simulations, we compared several ways to construct a GRS. If E exacerbates the adverse influence of a gene, GRS formed by the elastic net (ENET) or the least absolute shrinkage and selection operator (LASSO) is recommended. However, the performance of ENET or LASSO will be compromised if E attenuates the adverse influence of a gene, and using the ridge regression (RIDGE) can be more powerful in this situation. Applying RIDGE to 18,424 subjects in the Taiwan Biobank, we showed that performing regular exercise can attenuate the adverse influence of the *FTO* gene on four obesity measures: BMI (*p* = 0.0009), body fat percentage (*p* = 0.0031), waist circumference (*p* = 0.0052), and hip circumference (*p* = 0.0001). As another example, we used RIDGE and found the *FGF5* gene has a stronger effect on blood pressure in Han Chinese with a higher waist-to-hip ratio [*p* = 0.0013 for diastolic blood pressure (DBP) and *p* = 0.0027 for systolic blood pressure (SBP)]. This study provides an evaluation on the GRS approaches, which is important to infer whether E attenuates or exacerbates the adverse influence of a candidate gene.

## Introduction

The detection of “gene-environment interactions” (G × E) is important and is even more challenging than the detection of main effects of genes (Greenland, 1983; Hunter, 2005; Aschard, 2016). Although some gene-based G × E methods have been developed (Jiao et al., 2013; Lin et al., 2013; Chen et al., 2014; Lin et al., 2016; Lin et al., 2019), very few G × E findings have reached the genome-wide significance level (i.e., $p<\frac{0.05}{20,000}=2.5\times 10^{-6}$. Because there are ∼20,000 genes across the genome, 2.5 × 10^–6^ is the commonly used significance level in genome-wide gene-based analyses) (Chen et al., 2014; Lin et al., 2019). Most evidence of G × E was discovered by candidate gene analyses in which genome-wide association study (GWAS) hits were targeted. For example, physical activity has been found to attenuate the influence of the *fat mass and obesity-associated* (*FTO*) gene on obesity risk (Vimaleswaran et al., 2009; Kilpelainen et al., 2011). This means that the association of the *FTO* risk alleles with obesity measures is weaker in physically active subjects than in physically inactive subjects.

Another example is related to blood pressure levels. The *fibroblast growth factor 5* (*FGF5*) gene is associated with blood pressure levels in Han Chinese (Liu et al., 2011; Lin et al., 2019). Obesity has been reported to exacerbate the adverse influence of *FGF5* on blood pressure. That is, the association of the *FGF5* risk alleles with blood pressure is stronger in obese subjects than in lean subjects (Li et al., 2015).

A candidate gene usually harbors multiple risk variants. It is of interest to know whether an environmental factor (E) attenuates or exacerbates the effects of these risk variants. However, most G × E methods provide only *p*-values without any inference for the direction of interaction (Lin et al., 2013, 2016, 2019; Chen et al., 2014). In contrast, a genetic risk score (GRS) approach aggregates the effects among an ensemble of single-nucleotide polymorphisms (SNPs) and can indicate whether the GRS interacts synergistically or antagonistically with E. A GRS is a linear combination of risk allele counts, where risk alleles and weights are usually retrieved from large published GWASs or meta-analyses. However, the vast majority of genetic studies have been performed on subjects of European ancestry (Sirugo et al., 2019). Appropriate external GWAS results may not be available for G × E studies in other ethnic populations.

To address this issue, GRS approaches using internal weights have been proposed for pathway-based G × E studies (Huls et al., 2017; Huls et al., 2017) and GWASs (Lin et al., 2018, 2019, 2020). The internal weights come from marginal effects of SNPs that can be estimated by a multivariate elastic net (ENET) regression (Huls et al., 2017; Huls et al., 2017).

In this study, we described how to use GRS approaches to infer whether E attenuates or exacerbates the adverse influence of a gene (therefore, GRS here aggregates the effects among an ensemble of SNPs in a gene). Moreover, we compared GRS approaches with the “Set-Based gene-EnviRonment InterAction test” (SBERIA) (Jiao et al., 2013), the “interaction sequence kernel association test” (iSKAT) (Lin et al., 2016), and our previously developed “adaptive combination of Bayes factors method” (ADABF) (Lin et al., 2019). These methods were then applied to the Taiwan Biobank (TWB) data.

## Materials and Methods

### Genetic Risk Score Approaches

Usually, a GRS combines information of multiple nearly independent SNPs across the genome (Ahmad et al., 2013; Rask-Andersen et al., 2017; Lin et al., 2019, 2020). However, there have been few applications of using GRS in gene-based G × E tests. To have a clear description, we first explain covariate adjustment in the GRS gene-based G × E approach. Suppose there are *L* SNPs in a gene or a pathway. Let *g*[⋅] be the link function, *Y_i* be the phenotype that can be either continuous or binary, *G*_*ij*_ be the number of minor alleles (0, 1, or 2, by additive genetic model) at the *j*-th SNP (*j* = 1, …, *L*), *E_i* be the environmental factor, _**Xi**_ be the vector of non-genetic covariates such as the age and sex, and the subscript “*i*” represents data for the *i*-th subject (*i*=1,⋯,*n*). In addition to the additive model (by counting the number of minor alleles), *G*_*ij*_ can also be coded according to the dominant or recessive genetic model.

Some GRS approaches involve SNP selection and then aggregate the information of selected SNPs. Therefore, we leave *G*_*ij*_(*j*=1,…,*L*) later and first regress *Y_i* on _**Xi**_ by a linear model or a logistic regression model, as follows:

(1) $$g\,\left[E\,\left(Y_{i}\right)\right]=\alpha_{0}+\alpha^{\prime }\,{\text{X}}_{i},i=1,\ldots ,n.$$

Let $\hat{\mu_{0\,i}}=\hat{\alpha_{0}}+\hat{\alpha {}^{\prime }}\,{\text{X}}_{i}$ (for continuous *Y_i*) or $\hat{\mu_{0\,i}}=\frac{e\,x\,p\,\left(\hat{\alpha_{0}}+\hat{\alpha {}^{\prime }}\,X_{i}\right)}{1+e\,x\,p\,\left(\hat{\alpha_{0}}+\hat{\alpha {}^{\prime }}\,X_{i}\right)}$ (for binary *Y_i*) be the predicted mean of *Y_i* under model (1). Therefore, the covariate-adjusted phenotype for the *i*-th subject is $\hat{\varepsilon_{i}}=Y_{i}-\hat{\mu {}_{0\,i}}$.

Subsequently, we regress $\hat{\varepsilon_{i}}$ on *G*_*i*1_,⋯,*G*_*iL*_, as follows:

(2) $$g\,\left[E\,\left(\hat{\varepsilon_{i}}\right)\right]=\beta_{0}+\sum_{j=1}^{L}\beta_{j}\,G_{i\,j},i=1,\cdots ,n.$$

Let β′=[β_0_...β_*L*_] be the vector of regression coefficients in model (2). The ordinary least squares (OLS) estimate of β is as follows:

(3) β^=a⁢r⁢g⁢m⁢i⁢nβ⁢[∑i=1n(εi^-β0-∑j=1Lβj⁢Gi⁢j)2]=(G⁢G′)-1⁢(G⁢ε^′),(G⁢G′)-1⁢(G⁢ε^′),

where *n* is the sample size, **G** is the *n*×(*L*+1) matrix with the *i*-th row of [1*G*_*i*1_⋯*G*_*iL*_], and $\hat{\varepsilon }$ is the *n*-length vector of covariate-adjusted phenotypes. Because of linkage disequilibrium (LD), the SNPs in a gene are usually highly correlated with each other. In this situation, G⁢G′ may be singular and not invertible.

Ridge regression (RIDGE) (Hoerl and Kennard, 1970) is used to address the collinearity problem in model (2), where the regression coefficients are estimated by minimizing the residual sum of squares and an *l*_2_-norm penalty, as follows:

(4) β^=a⁢r⁢g⁢m⁢i⁢nβ[∑i=1n(εi^-β0-∑j=1LβjGi⁢j)2+λ∑j=1Lβj2].

The regularization parameter λ controls the amount of shrinkage. When λ is close to 0, $\hat{\beta }$ from RIDGE will approximate the $\hat{\beta }$ from OLS. When λ is large, $\hat{\beta_{1}},\cdots ,\hat{\beta_{L}}$ will approach 0, and model (2) will be reduced to an intercept-only model.

Least absolute shrinkage and selection operator (LASSO) was later proposed to estimate regression coefficients by minimizing the residual sum of squares and an *l*_1_-norm penalty (Tibshirani, 1996) as follows:

(5) β^=a⁢r⁢g⁢m⁢i⁢nβ[∑i=1n(εi^-β0-∑j=1LβjGi⁢j)2+λ∑j=1L|βj|].

The regularization parameter λ controls the amount of shrinkage. Moreover, variable selection can be performed by shrinking some β_*j*_*s* to 0 (*j*=1,…,*L*). Because equation (5) selects SNPs that are marginally associated with the covariate-adjusted phenotype ($\hat{\varepsilon_{i}},i=1,\cdots ,n$), this is called the marginal-association filtering by LASSO. The term “marginal” is used here because the *E_i* and *G*_*ij*_×*E*_*i*_ interaction term have not been included in Eq. (5). If β_*j*_ is shrunk to 0, the *j*-th SNP is regarded as unassociated with the covariate-adjusted phenotype and will not be used for the construction of a GRS.

ENET (Zou and Hastie, 2005) strikes a balance between RIDGE and LASSO by estimating regression coefficients while minimizing the residual sum of squares and a mixture of an *l*_1_-norm and an *l*_2_-norm, as follows:

(6) β^=a⁢r⁢g⁢m⁢i⁢nβ{∑i=1n(εi^-β0-∑j=1LβjGi⁢j)2+λ∑j=1L[12(1-α)βj2+α|βj|]}.

The regularization parameter λ controls the amount of shrinkage, whereas α is a penalty weight ranging from 0 (RIDGE) to 1 (LASSO). As suggested by Huls et al. (2017), α=0.5 is used for ENET throughout this work to achieve an optimal balance between RIDGE and LASSO. Eq. (6) is the marginal-association filtering by ENET.

Similar to genetic studies using penalized regression approaches (Waldmann et al., 2013; Huls et al., 2017; Huls et al., 2017), we used the R package “glmnet” (Friedman et al., 2010) to obtain $\hat{\beta }$. The optimal values of the regularization parameter λ in Eqs (4–6) were determined by 10-fold cross-validation. GWASs (Waldmann et al., 2013) and pathway-based G × E studies (Huls et al., 2017; Huls et al., 2017) have recommended choosing the largest λ such that the mean squared error (MSE) is within 1 standard error of the minimum MSE, to avoid selecting too many SNPs in ENET or LASSO. However, most complex traits are polygenic, and a single gene usually explains little phenotypic variation. In our simulations and real data analyses, this criterion usually selected 0 SNPs for ENET or LASSO. Therefore, in gene-based G × E studies, we recommend using the λ that leads to the minimum MSE.

RIDGE, LASSO, and ENET are all techniques for regression models that suffer from multicollinearity. Therefore, the pruning stage to remove SNPs with LD is not required here. After obtaining $\hat{\beta }$ from RIDGE, LASSO, or ENET, the GRS of the *i*-th subject is constructed by $G\,R\,S_{i}^{\prime }=\sum_{j=1}^{L}\hat{\beta_{j}}\,G_{i\,j}$, where *G*_*ij*_ is the number of minor alleles (0, 1, or 2) at the *j*-th SNPof the *i*-th subject. A positive $\hat{\beta_{j}}$ indicates that the minor allele is associated with an increase in phenotype. Therefore, a subject with more copies of the minor allele (more phenotype-increasing alleles) will obtain an increase in his/her *GRS*′. In contrast, a negative $\hat{\beta_{j}}$ indicates that the minor allele is associated with a decrease in phenotype, and a subject with more copies of the minor allele (more phenotype-decreasing alleles) will obtain a decrease in his/her *GRS*′. The *GRS*′ is then transformed into a *Z*-score that represents how many standard deviations the *GRS*′ is from the mean. The standardized GRS *Z*-score is denoted as *GRS*_*i*_. A larger GRS is always associated with an increased phenotype.

Afterwards, we fit the following generalized linear model (GLM):

(7) g⁢[E⁢(Yi)]=γ0+γG⁢G⁢R⁢Si+γE⁢Ei+γI⁢n⁢t⁢G⁢R⁢Si×Ei+γ′C⁢Xi,i=1,⋯,n,

where γ_*G*_ > 0 because a larger GRS is always associated with an increased phenotype. Adding a constraint (γ_*G*_ > 0) is expected to improve power, although for simplicity we here perform a GLM without this constraint. By testing *H*_0_:γ_*Int*_=0*vs*.*H*_1_:γ_*Int*_≠0, we evaluate whether G × E exists. For continuous traits, each 1 standard deviation (SD) increase in GRS is associated with a $\hat{\gamma_{I\,n\,t}}$ change in phenotype for subjects exposed to E = 1 than for subjects exposed to E = 0. For binary traits, each 1 SD increase in GRS is associated with an odds ratio of exp$\left(\hat{\gamma_{I\,n\,t}}\right)$ for subjects exposed to E = 1 compared to subjects exposed to E = 0. A positive (negative) $\hat{\gamma_{I\,n\,t}}$ indicates that E = 1, or a larger E, exacerbates (attenuates) the adverse influence of a candidate gene.

The whole data can be used to estimate β_1_,⋯,β_*L*_ (Eqs 4–6) and then to test *H*_0_:γ_*Int*_=0*vs*.*H*_1_:γ_*Int*_≠0 (model 7) without data splitting. Theoretically, $\hat{\beta_{j}}\left(j=1,\cdots ,L\right)$ and $\hat{\gamma_{I\,n\,t}}$ are asymptotically independent under the null hypothesis of no SNP-by-environment interaction, proved by corollary 1 of Dai et al. (2012). A two-stage approach that first filters SNPs by a criterion independent of the test statistic ($\hat{\gamma_{I\,n\,t}}$ estimated from model 7) under the null hypothesis, and then only uses SNPs that pass the filter, can maintain type I error rates and boost power (Bourgon et al., 2010; Frost et al., 2016). Empirically, our following simulation studies confirmed that GRS using internal weights is a valid approach in the sense that type I error rates match the nominal significance level. Moreover, studies on genes (Jiao et al., 2013; Frost et al., 2016), pathways (Huls et al., 2017; Huls et al., 2017), and GWASs (Lin et al., 2018) have presented the validity of using internally weighted GRSs to test for G × E.

### Competing Methods

The abovementioned GRS approaches with some form of penalized regression (RIDGE, LASSO, or ENET) were compared with the SBERIA (Jiao et al., 2013), iSKAT (Lin et al., 2016), and ADABF (Lin et al., 2019) methods.

SBERIA is the first gene-based G × E test based on the GRS concept. The phenotype is first regressed on each SNP separately, while adjusting for covariates such as sex, age, and ancestry principal components (PCs), as follows:

(8) $$g\,\left[E\,\left(Y_{i}\right)\right]=\beta_{0}+\beta_{j}\,G_{i\,j}+\beta_{C}^{{}^{\prime }}\,{\text{X}}_{i},i=1,\cdots ,n;j=1,\cdots ,L.$$

Let $\hat{\beta_{j}}$ be the estimated regression coefficient of the *j*-th SNP and *p_j* be the *p*-value of testing *H*_0_:β_*j*_≠0*vs*.*H*_1_:β_*j*_≠0. The GRS of the *i*-th subject is constructed by $GRS_{i}^{\prime }=\sum_{j=1}^{L}\left[I\left(p_{j}<0.1\right)sign\left(\hat{\beta_{j}}\right)+\nu \right]G_{i\,j}$, where *I*(*p*_*j*_ < 0.1) is an indicator variable with a value of 1 if *p*_*j*_ < 0.1 and 0 otherwise, $s\,i\,g\,n\,\left(\hat{\beta_{j}}\right)$ is either 1 or –1 depending on the sign of $\hat{\beta_{j}}$, and ν is a very small value (e.g., 0.0001). Therefore, SBERIA GRS includes both SNP selection (only SNPs marginally associated with the phenotype are used to construct the GRS) and SNP weighting (only the directions of how SNPs influence the phenotype are used in the GRS). The standardized GRS *Z*-score is denoted as *GRS*_*i*_. Then, the following GLM is fitted:

(9) g⁢[E⁢(Yi)]=γ0+∑j=1LγGj⁢Gi⁢j+γE⁢Ei+γI⁢n⁢t⁢G⁢R⁢Si×Ei+γ′C⁢Xi,i=1,⋯,n;j=1,⋯,L.

By testing *H*_0_:γ_*Int*_=0*vs*.*H*_1_:γ_*Int*_≠0, we can evaluate whether G × E exists (Jiao et al., 2013).

There are two fundamental differences between SBERIA and ENET (or LASSO). First, in the marginal-association filtering stage, SBERIA fits *L* regression models, respectively (Eq. 8), whereas ENET and LASSO fit a multivariate model incorporating *L* SNPs simultaneously (Eqs 5 and 6). Second, in the model for testing GRS × E, SBERIA includes the main effects of all the *L* SNPs ($\sum_{j=1}^{L}\gamma_{G_{j}}\,G_{i\,j}$ in Eq. 9), whereas ENET and LASSO incorporate only a GRS term as the aggregated genetic main effects (γ_*G*_*GRS*_*i*_ in Eq. 7).

We compared the abovementioned GRS tests with iSKAT (Lin et al., 2016) and ADABF (Lin et al., 2019). In iSKAT, the following model is considered:

(10) g⁢[E⁢(Yi)]=δ0+∑j=1LδGj⁢Gi⁢j+δE⁢Ei+∑j=1LδI⁢n⁢tj⁢Gi⁢j⁢Ei+δ′C⁢Xi,i=1,⋯,n,

where δ_*Int*_*j*__ is the interaction effect between the *j*-th SNP and E. Assuming δ_*Int*_*j*__*s*(*j*=1,⋯,*L*) follow a distribution with a mean of 0 and a variance of τ, the null hypothesis of all δ_*Int*_*j*__*s*=0(*j*=1,⋯,*L*) can be reduced to τ=0. The iSKAT is a score statistic for testing the variance component, i.e., *H*_0_:τ=0 vs. *H*_1_:τ > 0. The statistic can be referred to as Eq. (6) in Lin et al. (2016). The iSKAT method is regarded as optimal in the class of variance component tests (Lin et al., 2013; Chen et al., 2014). Therefore, we chose iSKAT as the representative of variance component tests.

In ADABF, we consider the following model for the *j*-th SNP (*j*=1,⋯,*L*):

(11) g⁢[E⁢(Yi)]=δ0+δGj⁢Gi⁢j+δE⁢Ei+δI⁢n⁢tj⁢Gi⁢j⁢Ei+δ⁢XiC′,i=1,⋯,n.

The SNP × E interaction is of interest, and therefore, *H*_0_:δ_*Int*_*j*__=0 vs. *H*_1_:δ_*Int*_*j*__≠0 for the *j*-th SNP (*j*=1,⋯,*L*). A Bayes factor (BF) was calculated for each SNP × E, where *BF*=*Pr*(*Data*|*H*_1_)/*Pr*(*Data*|*H*_0_). A larger BF indicates that the relative evidence in favor of *H_1* (SNP × E interaction exists) is stronger. Because the number of SNPs exhibiting interactions with E varies gene by gene, ADABF exhaustively searches for the evidence of G × E interaction by considering the largest BF, combining the largest 2 BFs, combining the largest 3 BFs,…, to aggregating all the *L* BFs in the gene. The significance of G × E interaction is then determined by the efficient sequential resampling procedure (Liu et al., 2016). ADABF was selected for comparison because it was recommended as a powerful and robust gene-based G × E test in a recent study (Lin et al., 2019). The iSKAT and ADABF methods do not test for G × E through a GRS term. Therefore, these two methods do not make inference for the direction of G × E.

### Simulation Study

To reflect the real LD structures of the human genome, we used the genotypes of 18,424 TWB subjects as our simulation material. Three genes (i.e., *TNNT3*, *RFX3*, and *FTO*) were drawn for simulations, including 48, 95, and 242 SNPs, respectively. It is common to see multiple trait-associated SNPs to be included in the same gene (Willer et al., 2007; Fawcett and Barroso, 2010). Therefore, we randomly specified 4 trait-associated SNPs in each simulation replicate. We considered binary and continuous exposures, respectively. For binary exposures, E was randomly sampled from 1 (exposed) or 0 (non-exposed), with *P* (*E* = 1) = 0.2 and *P* (*E* = 1) = 0.5, respectively. For continuous exposures, E was randomly selected from the normal distribution with a mean of 0 and a standard deviation of 0.5.

The continuous trait of the *i*-th subject was simulated as follows:

(12) $$Y_{i}=\sum_{d=1}^{4}\beta_{G_{d}}\,G_{i\,d}+\beta_{E}\,E_{i}+\sum_{d=1}^{D}\beta_{I\,n\,t_{d}}\,G_{i\,d}\,E_{i}+\varepsilon_{i},$$

where β_*G*__*d*_ is the SNP main effect of the *d*-th trait-associated SNP (*d*=1,⋯,4), β_*Int*_*d*__ is the effect size of interaction between the *d*-th trait-associated SNP and E (*d*=1,⋯,*D*), *D* is the number of trait-associated SNPs that also exhibit interactions with E (*D* = 4 or 2 in our simulation), and ε_*i*_ is the random error term following the standard normal distribution.

The binary trait of the *i*-th subject was simulated as follows:

(13) $$l\,o\,g\,\frac{P\left(Y_{i}=1\right)}{1-P\left(Y_{i}=1\right)}=\beta_{0}+\sum_{d=1}^{4}\beta_{G_{d}}\,G_{i\,d}+\beta_{E}\,E_{i}+\sum_{d=1}^{D}\beta_{I\,n\,t_{d}}\,G_{i\,d}\,E_{i},$$

where the intercept β_0_ was *log*(0.1/0.9)=−2.2 or *log*(0.4/0.6)=−0.4. *Y*_*i*_=1 represents that the *i*-th subject was diseased whereas *Y*_*i*_=0 indicates that he/she was non-diseased. This setting corresponds to a disease prevalence of 10% or 40%. A disease prevalence of 40% is also a reasonable setting for some complex diseases. For example, the worldwide prevalence of hypertension among adults aged ≥25 years was ∼40% (Abebe et al., 2015).

The magnitudes of SNP main effects, |β_*G*__*d*_|*s*(*d*=1,⋯,4), were uniformly sampled from 0.04 to 0.08 for continuous traits or uniformly sampled from *log*(1.05) to *log*(1.15) for binary traits. This simulation setting for binary trait (odds ratios uniformly sampled from 1.05 to 1.15) concurs with broad GWAS findings, where most odds ratios < 1.5 and many odds ratios < 1.2 (Baxter and Jordan, 2012; Hodge and Greenberg, 2016).

Unlike small effect sizes for SNPs, the effect size of E was assumed to be larger, because some E is critical to traits (e.g., regular exercise and dietary habits are important to obesity measures). The magnitude of E, |β_*E*_|, was assigned to be 0.3 for continuous traits and *log*(1.3)=0.2624 for binary traits, respectively. When evaluating type I error rates, data have to be generated from the null hypothesis of no *SNP*×*E* interactions, and therefore we specified all β_*Int*_*d*__*s*=0 (*d*=1,⋯,*D*). When assessing power, the magnitudes of *SNP*×*E* interaction effects, |β_*Int*_*d*__|*s* (*d*=1,⋯,*D*), were uniformly sampled from 0.04 to 0.08 for continuous traits or uniformly sampled from *log*(1.05) to *log*(1.15) for binary traits. To not favor GRS-based approaches that construct GRSs with SNPs marginal effects, the sampling of |β_*Int*_*d*__|*s* (*d*=1,⋯,*D*) was independent of the sampling of |β_*G*__*d*_|*s* (*d*=1,⋯,4). Power was evaluated under 14 simulation scenarios listed in Table 1, in which “+” indicates a positive effect and “−” means a negative effect. As summarized by Table 1, we always assumed 4 trait-associated SNPs, but the number of trait-associated SNPs that also exhibited interactions with E could be 2 or 4. This setting mimics the common situation that some trait-associated SNPs may also present interactions with E (Jonsson et al., 2009; Chen et al., 2014; Li et al., 2015).

**TABLE 1:** The 14 simulation scenarios for power comparison, where “exacerbation” and “attenuation” mean that *E* = 1 (or a larger continuous *E*) exacerbates or attenuates the adverse effect of a candidate gene, respectively.

Scenario	*E*	β_G1_	β_G2_	β_G3_	β_G4_	β_Int1_	β_Int2_	β_Int3_	β_Int4_
1 Exacerbation	+	+	+	+	+	+	+	+	+
2 Attenuation	+	+	+	+	+	−	−	−	−
3 Exacerbation	+	+	+	+	+	+	+	0	0
4 Attenuation	+	+	+	+	+	−	**−**	0	0
5 Cross-over	+	+	+	+	+	+	+	−	−
6 Exacerbation	+	+	+	−	−	+	+	−	−
7 Attenuation	+	+	+	−	−	−	−	+	+
8 Exacerbation	−	+	+	+	+	+	+	+	+
9 Attenuation	−	+	+	+	+	−	−	−	−
10 Exacerbation	−	+	+	+	+	+	+	0	0
11 Attenuation	−	+	+	+	+	−	−	0	0
12 Cross-over	−	+	+	+	+	+	+	−	−
13 Exacerbation	−	+	+	−	−	+	+	−	−
14 Attenuation	−	+	+	−	−	−	−	+	+

Scenarios 1–7 describe that a larger E is associated with an increase in trait, whereas scenarios 8–14 represent that a larger E is associated with a decrease in trait. Suppose that a higher trait is linked to a less healthy situation, e.g., *Y*_*i*_=1 in binary traits. Scenarios 1, 3, 6, 8, 10, and 13 indicate that a larger E exacerbates the adverse effect of a candidate gene, whereas scenarios 2, 4, 7, 9, 11, and 14 present that a larger E attenuates the adverse effect of a candidate gene. Scenarios 5 and 12 are cross-over situations, representing that a larger E exacerbates the adverse effect of 50% of trait-associated SNPs while attenuating the adverse effect of the remaining 50% of trait-associated SNPs.

### Application to the Taiwan Biobank Data

The TWB aims to build a research database that integrates the genomic profiles and lifestyle patterns of residents aged 30–70 years in Taiwan (Chen et al., 2016). Community-based volunteers provided blood samples and a range of information via a face-to-face interview and physical examination. This study included 20,287 TWB individuals and was approved by the Research Ethics Committee of the National Taiwan University Hospital (NTUH-REC No. 201805050RINB). To remove cryptic relatedness, we used PLINK 1.9 (Purcell et al., 2007) to calculate the genome-wide identity by descent (IBD) sharing coefficients between any two subjects. Similar to many genetic studies (Lowe et al., 2009; Mok et al., 2014; Ombrello et al., 2014), we excluded one subject from each pair with PI-HAT ≥ 0.125, where PI-HAT = Probability (IBD = 2) + 0.5 × Probability (IBD = 1). Through this step, relatives within the third-degree of consanguinity were removed. Finally, 18,424 unrelated subjects (9,093 males and 9,331 females) were remained in the following analysis. Table 2 shows basic characteristics of TWB participants stratified by sex.

**TABLE 2:** Basic characteristics of TWB participants stratified by sex.

	Overall	Males	Females
Total, *n* (%)	18,424	9,093	9,331
Age (years), mean (SD)	48.9 (11.0)	49.0 (11.0)	48.9 (10.9)
Smoking, *n* (%)	2,134(11.6)	1,882(20.7)	252 (2.7)
Drinking, *n* (%)	1,345(7.3)	1,178(13.0)	167 (1.8)
Regular exercise, *n* (%)	7,652(41.5)	3,896(42.8)	3,756(40.3)
Educational attainment, mean (SD)	5.46 (0.99)	5.62 (0.92)	5.29 (1.02)
BMI (kg/m^2^), mean (SD)	24.31 (3.66)	25.2 (3.4)	23.5 (3.7)
Body fat %, mean (SD)	27.29 (7.38)	22.9 (5.5)	31.5 (6.5)
Waist circumference (cm), mean (SD)	83.93 (10.03)	87.4 (9.1)	80.5 (9.7)
Hip circumference (cm), mean (SD)	96.34 (6.90)	97.6 (6.5)	95.2 (7.0)
Waist-hip ratio, mean (SD)	0.87 (0.068)	0.90 (0.06)	0.85 (0.07)
Diastolic blood pressure (mmHg), mean (SD)	73.11 (11.10)	76.9 (10.6)	69.4 (10.3)
Systolic blood pressure (mmHg), mean (SD)	117.62 (17.37)	121.9 (16.1)	113.5 (17.6)

The majority of TWB subjects were of Han Chinese ancestry (Chen et al., 2016). The Axiom Genome-Wide TWB genotyping array was designed for Han Chinese in Taiwan, which was run on the Axiom Genome-Wide Array Plate System (Affymetrix, Santa Clara, CA, United States). A total of 646,783 autosomal SNPs were genotyped in this TWB array. After removing 51,293 SNPs with genotyping rates <95%, 6,095 SNPs with Hardy-Weinberg test *p*-values < 5.7 × 10^–7^ (WTCCC, 2007), and 1,869 variants with minor allele frequencies (MAFs) <1%, 587,526 SNPs were used to construct ancestry PCs. Because variants with MAF <1% have been excluded, no rare variants were included in our following analyses. Removing variants with MAFs <1% is a commonly used quality control step in genetic association studies, because the chances of errors in genotype calling increase with decreasing MAFs (Goldstein et al., 2012; Coleman et al., 2016).

## Results

### Type I Error Rates

**FIGURE 1:**
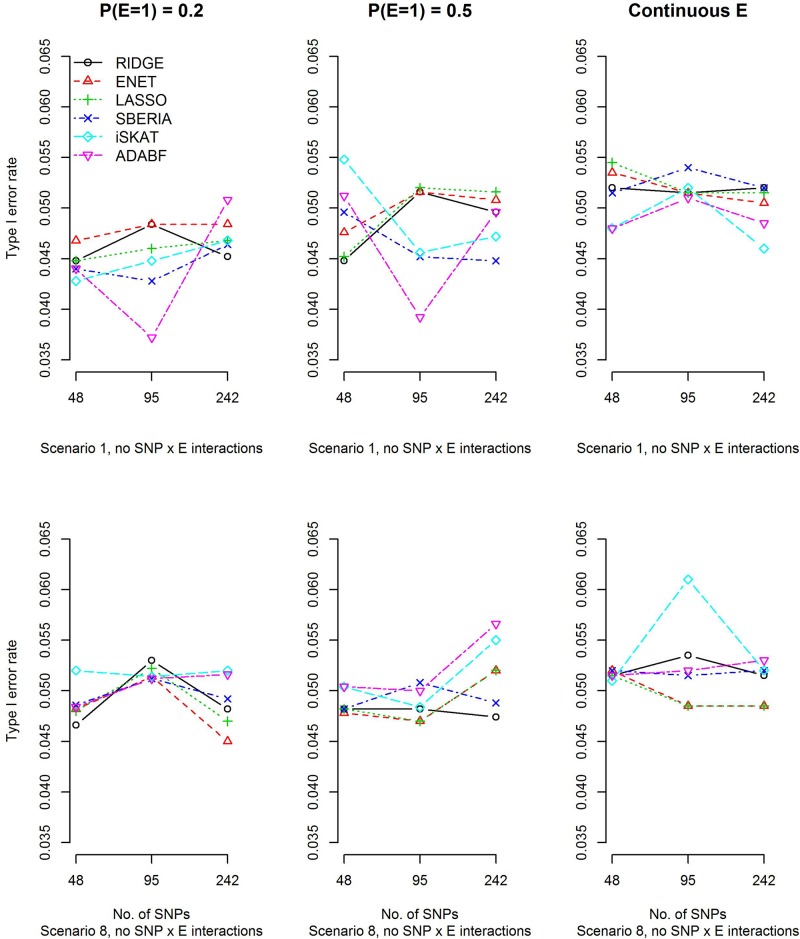
Empirical type I error rates under the nominal significance level of 0.05 (continuous trait).

To evaluate type I error rates, continuous traits and binary traits were simulated according to models (12) and (13), respectively. Scenarios 1 and 8 in Table 1 were simulated, but all β_*Int*_*d*__s (*d*=1,⋯,4) have to be set at 0 in order to evaluate type I error rates. The main effects of SNPs, |β_*G*__*d*_|s (*d*=1,⋯,4), and the environmental factor, |β_*E*_|, have been described in section “Simulation study.” Based on 10,000 replications for each scenario, Figure 1 and Supplementary Figure S1 show that all methods were valid in the sense that their type I error rates matched the nominal significance level.

### Power of 6 G × E Methods

**FIGURE 2:**
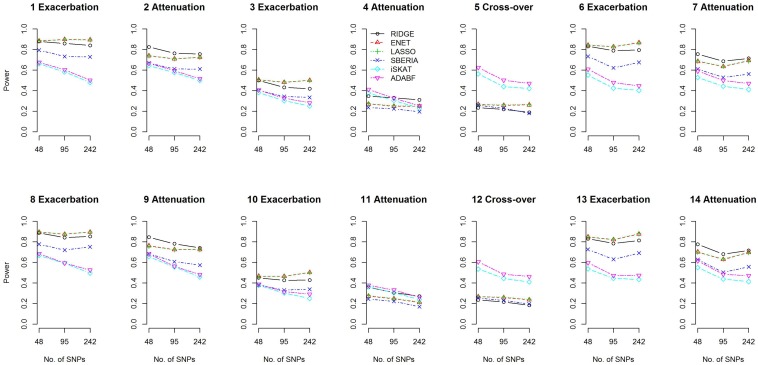
Power given a significance level of 0.05, for continuous traits and *P* (*E* = 1) = 0.2.

We compared the power of the 4 GRS-based tests and 2 G × E methods: iSKAT (Lin et al., 2016) and ADABF (Lin et al., 2019). The power of the 4 GRS-based tests is defined as the probability of rejecting *H*_0_:γ_*Int*_=0 (*p*-value < 0.05) and correctly specifying the sign of γ_*Int*_ (γ_*Int*_ can be found from models 7 and 9). The iSKAT (Lin et al., 2016) and ADABF (Lin et al., 2019) methods can only provide a *p*-value for testing G × E, without an inference of how E modifies the genetic effects. Therefore, their power is defined as the probability of rejecting the null hypothesis of no G × E (*p*-value < 0.05). Figure 2 presents the power based on 1,000 simulation replications under each scenario, for continuous traits and *P* (*E* = 1) = 0.2. If E exacerbates the adverse effect of a gene, ENET and LASSO outperform the other methods (scenarios 1, 3, 6, 8, 10, and 13). If E attenuates the adverse effect of a gene, RIDGE is more powerful (scenarios 2, 4, 7, 9, 11, and 14). Moreover, ADABF was the optimal test under the cross-over scenario (scenarios 5 and 12).

Among the 4 GRS-based tests, ENET, LASSO, and SBERIA first select SNPs marginally associated with the phenotype. ENET and LASSO select SNPs according to the multivariate ENET regression (Eq. 6) and multivariate LASSO regression (Eq. 5), respectively. SBERIA selects SNPs based on regressions considering one SNP at a time (Eq. 8). The performance of these three methods to detect G × E depends on their ability to find the true trait-associated SNPs. Supplementary Figures S2, S3 summarize the sensitivity (SEN) and positive predictive value (PPV) of the marginal-association filtering in ENET, LASSO, and SBERIA. As described in Eq. (12) and Table 1, 4 trait-associated SNPs were randomly assigned in each simulation replication. SEN is defined as the percentage of true positives among the 4 trait-associated SNPs, whereas PPV is defined as the percentage of true positives among the total findings.

Because SBERIA takes a liberal *p*-value threshold of 0.1, in the filtering stage it usually selects more SNPs than ENET and LASSO do. Therefore, as shown in Supplementary Figures S2, S3, SBERIA generally has a higher SEN but a lower PPV, compared with ENET and LASSO. For each method, SEN was much lower in attenuation scenarios than in exacerbation scenarios. This means that pinpointing true trait-associated SNPs is more difficult in attenuation scenarios. In the filtering stage (Eqs 5, 6, and 8), *G*_*id*_×*E*_*i*_ cannot be included; therefore, a β_*Int*_*d*__ in the opposite direction to β_*G*__*d*_ will weaken the magnitude of the marginal effect of the *d*-th trait-associated SNP. In contrast, a β_*Int*_*d*__ in the same direction to β_*G*__*d*_ will strengthen the magnitude of the marginal effect of the *d*-th trait-associated SNP. Therefore, for each method, the ability to pinpoint true trait-associated SNPs is inferior in attenuation scenarios than in exacerbation scenarios (as shown in Supplementary Figures S2, S16 for continuous traits and binary traits, respectively).

Because there is no SNP selection in RIDGE, true trait-associated SNPs are all reserved for constructing GRS. Therefore, RIDGE is the optimal GRS-based test in attenuation scenarios (Figure 2). In exacerbation scenarios, ENET and LASSO are the best two methods because of their superior ability in finding trait-associated SNPs (Supplementary Figures S2, S16).

**FIGURE 3:**
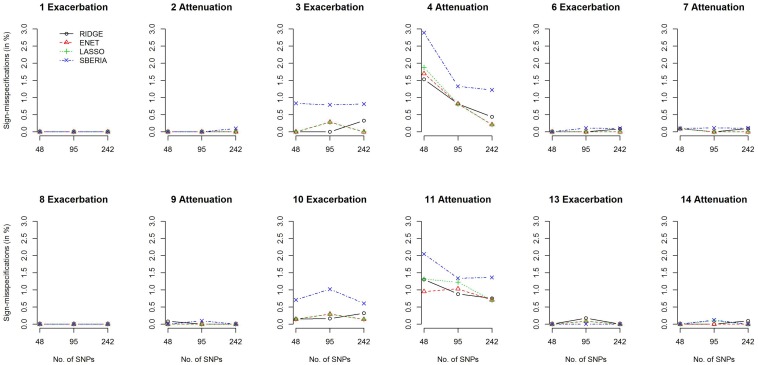
Percentages of sign-misspecifications for γ_*Int*_, under continuous traits and *P* (*E* = 1) = 0.2.

We then investigated the percentage of sign-misspecifications for each GRS-based method. In the presence of G × E, we calculated the percentage of wrongly specifying the sign of γ_*Int*_ (in models 7 or 9) among all rejections of *H*_0_:γ_*Int*_=0 (*p*-value < 0.05). With 1,000 simulation replications under each scenario, Figure 3 presents the percentages of sign-misspecifications, for continuous traits and *P* (*E* = 1) = 0.2. The true γ_*Int*_ is positive under scenarios 1, 3, 6, 8, 10, and 13; negative under scenarios 2, 4, 7, 9, 11, and 14. The cross-over scenarios (5 and 12) were not considered here, because the true sign of γ_*Int*_ was unclear when E = 1 (or a larger continuous E) exacerbates the adverse effect of 50% of trait-associated SNPs but attenuates the adverse effect of the remaining 50% of trait-associated SNPs.

Figure 3 shows that the signs of γ_*Int*_ were usually correctly specified except scenarios 4 and 11. SBERIA generally makes more mistakes in indicating the true direction for γ_*Int*_, because it filters SNPs by considering only one SNP at a time (Eq. 8). Supplementary Figures S4–S7 present the power and percentages of sign-misspecifications in continuous-trait simulations, given *P* (*E* = 1) = 0.5 and a continuous E, respectively. Each method under *P* (*E* = 1) = 0.5 was slightly more powerful than that under *P* (*E* = 1) = 0.2, but the comparisons across methods were similar to those shown in Figures 2, 3.

The performance of each method for binary-trait simulations can be found in Supplementary Figures S8–S21. Regarding different prevalence values, each method was more powerful under *P* (*Y* = 1) = 0.4 than under *P* (*Y* = 1) = 0.1. Concerning different probabilities of being exposed, each method was more powerful under *P* (*E* = 1) = 0.5 than under *P* (*E* = 1) = 0.2. Nonetheless, the comparisons across methods were similar to those described for continuous traits.

Regarding the 14 simulation scenarios listed in Table 1, only 2 SNP × E interactions (rather than 4) were simulated in scenarios 4 and 11, and β_*Int*_s were in the opposite direction to β_*G*_*s*. Therefore, detecting G × E and correctly specifying the sign for γ_*Int*_ were more challenging under scenarios 4 and 11.

Regarding the computation time (Supplementary Figures S22–S29), SBERIA is the fastest method, followed by the three penalized regression methods: RIDGE, ENET, and LASSO. ADABF uses the sequential resampling procedure (Liu et al., 2016), and therefore, it takes a longer time in the presence of G × E. A recent study concluded that ADABF is a computationally feasible method in a GWAS context (Lin et al., 2019). This is because in a real GWAS usually very few genes can be detected to interact with E. Therefore, a genome-wide analysis using ADABF takes a much shorter time than the power evaluation performed here (power is always evaluated in the presence of G × E). On average, the iSKAT method requires the longest time in most situations (Supplementary Figures S22–S29).

### *FTO* × Exercise Interaction on Five Obesity Measures

The *FTO* gene is obesity-associated and has been replicated by many studies (Frayling et al., 2007; Scuteri et al., 2007; Fawcett and Barroso, 2010). It spans from 53, 737, 875 to 54, 148, 379 base pairs on chromosome 16, according to the human genome GRCh37/hg19 assembly. Similar to many gene-based tests (Liu et al., 2010; Wray et al., 2012; Alonso-Gonzalez et al., 2019; Lin et al., 2019), 50 kb in the 3′ and 5′ regions that might regulate a gene were also incorporated in our analysis. Specifying a large boundary would be difficult to pinpoint the exact gene interacting with E, whereas a small boundary may not fully capture the regulatory regions (Liu et al., 2010). A total of 242 SNPs (all with MAF > 1%) were included in this chromosomal region. Here, we used the 6 G × E tests to investigate whether the influence of *FTO* can be modified by performing regular physical exercise. A total of five obesity measures were analyzed: body mass index (BMI), body fat percentage (BFP), waist circumference (WC), hip circumference (HC), and the waist-to-hip ratio (WHR).

Regular physical exercise was defined as engaging in 30 min of “exercise” three times a week. “Exercise” indicated leisure-time activities such as jogging, yoga, mountain climbing, or playing basketball. According to the TWB questionnaire, activities during work were not counted in “exercise.” Among the 18,424 subjects, 7,652 (41.5%) reported performing regular exercise, while 10,764 reported no regular exercise. A total of eight subjects did not respond to this question. Covariates adjusted in all models included sex, age (in years), educational attainment, drinking status, smoking status, and the first 10 ancestry PCs.

Table 3 presents the results of six methods. We may miss possibly important findings due to a harsh penalty of multiple testing. Because this study focuses on G × E detection for candidate genes, we set the significance level at 0.05. The results of RIDGE show that regular physical exercise attenuates the adverse effect of the *FTO* gene on BMI, BFP, WC, and HC. The other three GRS-based tests (ENET, LASSO, and SBERIA) provided significant results for three obesity measures, respectively. All $\hat{\gamma_{I\,n\,t}}$s were negative, indicating that regularly performing exercise attenuates the adverse influence of *FTO* on obesity measures.

**TABLE 3:** *FTO* × exercise interaction on five obesity measures.

Trait		RIDGE	ENET	LASSO	SBERIA	iSKAT	ADABF
BMI (kg/m^2^)	$\hat{\gamma_{I\,n\,t}}$	–0.1743	–0.0821	–0.0964	–0.1482		
	*P*_*Int*_	**0.0009**	0.1192	0.0671	**0.0067**	0.2043	0.1700
Body fat %	$\hat{\gamma_{I\,n\,t}}$	–0.2661	–0.2069	–0.2081	–0.2259		
	*P*_*Int*_	**0.0031**	**0.0212**	**0.0205**	**0.0160**	0.2430	0.2200
Waist circumference (cm)	$\hat{\gamma_{I\,n\,t}}$	–0.3854	–0.3719	–0.3760	–0.2786		
	*P*_*Int*_	**0.0052**	**0.0069**	**0.0063**	0.0512	0.5369	0.3700
Hip circumference (cm)	$\hat{\gamma_{I\,n\,t}}$	–0.3868	–0.3286	–0.3291	–0.2902		
	*P*_*Int*_	**0.0001**	**0.0011**	**0.0011**	**0.0055**	0.5061	0.3300
Waist-to-hip ratio	$\hat{\gamma_{I\,n\,t}}$	–0.000116	–0.000775	–0.000374	–0.000314		
	*P*_*Int*_	0.8951	0.3773	0.6699	0.7308	0.7994	0.3100

*Significant results with *P*_*Int*_ < 0.05 are highlighted in bold values. Negative $\hat{\gamma_{I\,n\,t}}$s sindicate that regular physical exercise attenuates the adverse influence of *FTO* on obesity measures.*

Genetic risk score-based tests are more powerful than iSKAT and ADABF because performing regular exercise generally blunted the effects of the trait-increasing alleles in *FTO*. Take BMI-associated SNPs as examples. A total of 12 out of the 242 SNPs reached the genome-wide significance (*p*-value < 5 × 10^–8^) when testing *H*_0_:β_*j*_=0*vs*.*H*_1_:β_*j*_≠ 0 in the following model:

(14) $$E\,\left(Y_{i}\right)=\beta_{0}+\beta_{j}\,G_{i\,j}+\beta_{C}^{{}^{\prime }}\,{\text{X}}_{i},$$

Where *Y_i* is BMI, *G*_*ij*_ is the number of minor alleles (0, 1, or 2) at the *j*-th SNP (*j* = 1, …, 242), and **X**_i_ is the vector of covariates of the *i*-th subject (*i*=1,⋯,18,424). All $\hat{\beta_{j}}$s of the 12 genome-wide significant SNPs were positive, representing that their minor alleles were associated with increased BMIs.

When exercise (*E*_*i*_=1 if yes; *E*_*i*_=0 if no) and the SNP × exercise interaction were included in the 12 models, as follows:

(15) $$E\,\left(Y_{i}\right)=\beta_{0}+\beta_{j}\,G_{i\,j}+\beta_{E}\,E_{i}+\beta_{I\,n\,t\,j}\,G_{i\,j}\times E_{i}+\beta_{C}^{{}^{\prime }}\,{\text{X}}_{i},j=1,\cdots ,12,$$

all $\hat{\beta_{j}}$s were positive, and all $\hat{\beta_{I\,n\,t\,j}}$s were negative (*j*=1,⋯,12). This indicated that the minor alleles of the 12 SNPs were associated with increased BMIs, but their effects were blunted by performing regular exercise. As mentioned above, a β_*Intj*_ in the opposite direction to β_*j*_ weakens the magnitude of marginal effect of this SNP, and therefore $\hat{\beta_{j}}$ from model (15) >$\hat{\beta_{j}}$ from model (14). This is a situation similar to our simulation scenario 9 in Table 1, because exercise is associated with a decrease in obesity measures. According to Figure 2 (scenario 9, 242 SNPs), RIDGE is the most powerful test. In real data, the *FTO* gene harbors more BMI-associated SNPs than our simulation scenario 9, and these SNPs interact with E in the same direction. Therefore, GRS-based tests are more powerful than iSKAT and ADABF.

**FIGURE 4:**
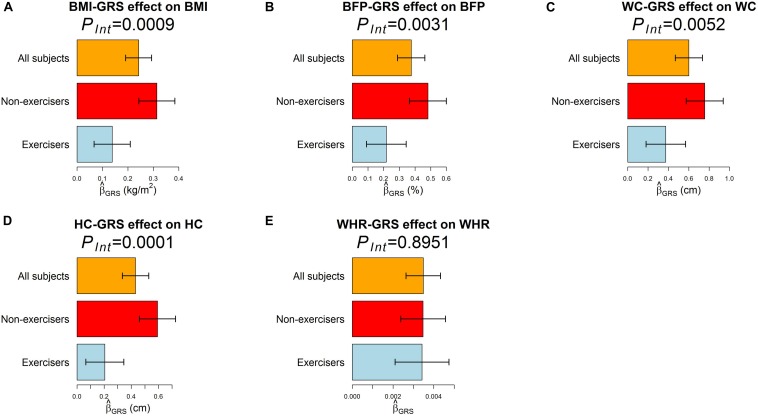
The effect of GRS on the five obesity measures. The regression model was built as *Obesitymeasure*=β_0_+β_*GRS*_*GRS*+β_***C***_**Covariates**+ε, where *GRS* was obtained by RIDGE. **(A–E)** are results for BMI, BFP, WC, HC, and WHR, respectively. Three regression models were built for each obesity measure, one for all 18,424 subjects, one for 7,652 exercisers, and one for 10,764 non-exercisers. The bars represent ${\hat{\beta }}_{G\,R\,S}$ on an obesity measure, and the black segments mark the 95% confidence intervals, i.e., $\left[{\hat{\beta }}_{G\,R\,S}-1.96\times \mathrm{standard}\,\mathrm{error}\,\mathrm{of}\,{\hat{\beta }}_{G\,R\,S},{\hat{\beta }}_{G\,R\,S}+1.96\times \mathrm{standard}\,\mathrm{error}\,\mathrm{of}\,{\hat{\beta }}_{G\,R\,S}\right]$. Covariates adjusted in all models included sex, age (in years), educational attainment, drinking status, smoking status, and the first 10 ancestry PCs.

According to RIDGE, each 1 SD increase in BMI-GRS was associated with a 0.1743 kg/m^2^ lower BMI in exercisers than in non-exercisers (*p* = 0.0009, Table 3). Each 1 SD increase in BFP-GRS was associated with a 0.2661% lower BFP in exercisers than in non-exercisers (*p* = 0.0031). Each 1 SD increase in WC-GRS was associated with a 0.3854 cm lower WC in exercisers than in non-exercisers (*p* = 0.0052). Each 1 SD increase in HC-GRS was associated with a 0.3868 cm lower HC in exercisers than in non-exercisers (*p* = 0.0001). As shown by Figure 4, except WHR, GRS effect on each obesity measure was smaller in exercisers than in non-exercisers. For each obesity measure, $\hat{\gamma_{I\,n\,t}}$ in Table 3 is approximately equal to the length of blue bar – the length of red bar in Figure 4. In the whole study population, each 1 SD increase in BMI-GRS was associated with a 0.2420 kg/m^2^ (the length of orange bar in Figure 4A) higher BMI (*P* = 1.1×10^−20^). This association was stronger in non-exercisers than in exercisers (*P*_*Int*_ = 0.0009, Table 3). In non-exercisers, each 1 SD increase in BMI-GRS was associated with a 0.3136 kg/m^2^ (the length of red bar in Figure 4A) higher BMI (*P* = 3.4×10^−18^); in exercisers, each 1 SD increase in BMI-GRS was associated with a 0.1382 kg/m^2^ (the length of blue bar in Figure 4A) higher BMI (*P* = 1.4×10^−4^).

Performing regular exercises is associated with an attenuation of the adverse influence of the *FTO* gene on both WC and HC, but not on WHR because it is a ratio of WC to HC. This negative result for WHR is in line with the result from polygenic analyses (i.e., aggregating information of multiple nearly independent SNPs across the genome) (Lin et al., 2019).

### *FGF5* × WHR Interaction on Blood Pressure Levels

In addition to binary exposures, we also provide an example for continuous exposures. The *FGF5* gene is associated with blood pressure levels in Han Chinese (Liu et al., 2011; Lin et al., 2019). *FGF5* spans from 81, 187, 742 to 81, 212, 171 base pairs on chromosome 4, according to the human genome GRCh37/hg19 assembly. Similar to many gene-based tests (Liu et al., 2010), our analysis region also included 50 kb in the 3′ and 5′ regions that might regulate the gene. A total of 38 SNPs (all with MAF >1%) were included in this chromosomal region. It has been known that central obesity is a risk factor for hypertension (Parrinello et al., 1996). Therefore, we used the 6 G × E tests to investigate whether the influence of *FGF5* can be modified by an indicator of central obesity, WHR. Covariates adjusted in all models included sex, age (in years), drinking status, smoking status, and the first 10 ancestry PCs. As described above, a total of 18,424 unrelated TWB subjects were included in our analysis, where 9,093 were males and 9,331 were females.

Table 4 presents the results of six methods. All six tests suggested the significance of *FGF5* × WHR interaction on DBP and SBP (*P*_*Int*_ < 0.05). Positive $\hat{\gamma_{I\,n\,t}}$s from GRS-based tests represent that a higher WHR exacerbates the adverse influence of *FGF5*.

**TABLE 4:** *FGF5* × WHR interaction on blood pressure levels.

Trait		RIDGE	ENET	LASSO	SBERIA	iSKAT	ADABF
DBP (mmHg)	$\hat{\gamma_{I\,n\,t}}$	0.2419	0.1980	0.2141	0.2378		
	*P*_*Int*_	**0.0013**	**0.0082**	**0.0042**	**0.0014**	**0**.**0154**	**0**.**0096**
SBP (mmHg)	$\hat{\gamma_{I\,n\,t}}$	0.3396	0.3548	0.3551	0.3261		
	*P*_*Int*_	**0.0027**	**0.0017**	**0.0017**	**0.0039**	**0**.**0482**	**0**.**0480**

*Significant results with *P*_*Int*_ < 0.05 are highlighted in bold values. Positive $\hat{\gamma_{I\,n\,t}}$s sindicate that a higher WHR exacerbates the adverse influence of *FGF5* on blood pressure levels.*

The above two examples demonstrate using GRS-based methods to infer whether E attenuates or exacerbates the adverse influence of a candidate gene (*FTO* and *FGF5*, respectively).

## Discussion

Detecting G × E has been an important but challenging issue. Although set-based G × E tests have been evaluated in a genome-wide context, very few genes have been found to interact with some exposures at the genome-wide significance level (i.e., $p<\frac{0.05}{20000}=2.5\times 10^{-6}$. Because there are ∼20,000 genes across the genome, 2.5×10^−6^ is the commonly used significance level in genome-wide gene-based analyses) (Chen et al., 2014; Lin et al., 2019). Searching for evidence of G × E for candidate genes may still be a more feasible strategy.

It is common to see a GRS that aggregates information of multiple nearly independent SNPs across the genome (Ahmad et al., 2013; Rask-Andersen et al., 2017; Lin et al., 2019, 2020). However, there have been few applications of using GRS in gene-based G × E tests. In this work, we evaluate the performance of GRS gene-based G × E approach, with simulations and real applications. GRS-based tests can outperform other methods if interactions tend to go in the same direction (Aschard, 2016). The filtering stage of SBERIA overlooks LD among SNPs because it considers *L* SNPs in *L* respective regressions (Eq. 8). RIDGE has the advantage of accommodating LD among SNPs and providing regression coefficients that lead to minimum MSE. Although RIDGE has been used to address LD in genetic association analysis (Malo et al., 2008), our study is the first attempt to use the ridge regression to construct a GRS for G × E analyses. ENET and LASSO can not only accommodate LD among SNPs, but they also select SNPs by shrinking some regression coefficients to 0.

Although ENET has been suggested as a powerful G × E test in pathway analyses (Huls et al., 2017; Huls et al., 2017), in this study, we showed that its performance to find trait-associated SNPs can be compromised if E attenuates the adverse effect of a gene (simulation scenarios 2, 4, 7, 9, 11, and 14). As a result, RIDGE is recommended if E attenuates the adverse effects of most SNPs in a gene, as shown in the application of *FTO* × exercise interaction on obesity measures (Table 3). In contrast, if E exacerbates the adverse effects of most SNPs in a gene, ENET and LASSO will be more powerful, as shown in the application of *FGF5* × WHR interaction on SBP (Table 4).

In this work, we described how to use GRS approaches to infer whether E attenuates or exacerbates the adverse effect of a gene. GRS approaches can not only provide a *p*-value for G × E but also infer how E interacts with the gene. The signs of γ_*Int*_ were usually correctly specified except scenarios 4 and 11, where only 2 SNP × E interactions were specified and their interaction effects were in the opposite direction to the SNP main effects. SBERIA usually makes more mistakes because its filtering stage overlooks LD among SNPs by considering *L* SNPs in *L* respective regressions (Eq. 8).

In the cross-over situations (simulation scenarios 5 and 12), where E exacerbates the adverse effects of some SNPs but attenuates the adverse effects of others, GRS approaches were underpowered and ADABF became the most useful. None of the six methods could outperform the others across all situations. In this work, we performed simulations to explore the relative performances of these six tests in detecting G × E and correctly indicating the interaction direction.

To show the utility of GRS-based approaches, we provided an example for binary exposure and an example for continuous exposure: (1) *FTO* × exercise interaction on five obesity measures (Table 3); (2) *FGF5* × WHR interaction on blood pressure levels (Table 4). The γ_*Int*_s in Table 3 were consistently negative, representing that performing regular exercise attenuates the adverse influence of the *FTO* gene on four obesity measures. Moreover, γ_*Int*_s in Table 4 were consistently positive, indicating that a higher WHR exacerbates the adverse effect of the *FGF5* gene on blood pressure levels.

This work provides contributions to an important issue: identify whether E attenuates or exacerbates the adverse influence of a candidate gene. Genes exhibiting interactions with E are usually difficult to detect and replicate (McAllister et al., 2017), especially at the genome-wide significance level (2.5×10^−6^). When testing for many genes simultaneously, even true positive G × Es would have difficulty in standing out among all the noise (Lin and Lee, 2010). Therefore, our discussions here are restricted to candidate genes. As TWB keeps recruiting more subjects, in the future we look forward to performing genome-wide G × E analyses on a larger TWB cohort.

## Data Availability Statement

Individual-level Taiwan Biobank data are available upon application to Taiwan Biobank (https://www.twbiobank.org.tw/new_web/).

## Ethics Statement

The studies involving human participants were reviewed and approved by the Research Ethics Committee of the National Taiwan University Hospital (NTUH-REC No. 201805050RINB). The patients/participants provided their written informed consent to participate in this study.

## Author Contributions

W-YL developed the methods and the analysis tools, designed and performed the simulation studies, analyzed the TWB data, and wrote the manuscript. Y-SL contributed to the programming for the simulations and data analyses. C-CC, Y-LL, S-JT, and P-HK contributed to the writing of the manuscript. P-HK, Y-LL, and S-JT provided the TWB data. All authors reviewed the manuscript.

## Conflict of Interest

The authors declare that the research was conducted in the absence of any commercial or financial relationships that could be construed as a potential conflict of interest.
