# Supplementary material for: Using Genetic Risk Score Approaches to Infer Whether an Environmental Factor Attenuates or Exacerbates the Adverse Influence of a Candidate Gene
Source: Front Genet. 2020 May 8;11:331. doi: 10.3389/fgene.2020.00331 (PMC7225361; doi:10.3389/fgene.2020.00331)
Supplement: Supplementary file 1 [file Data_Sheet_1.docx]

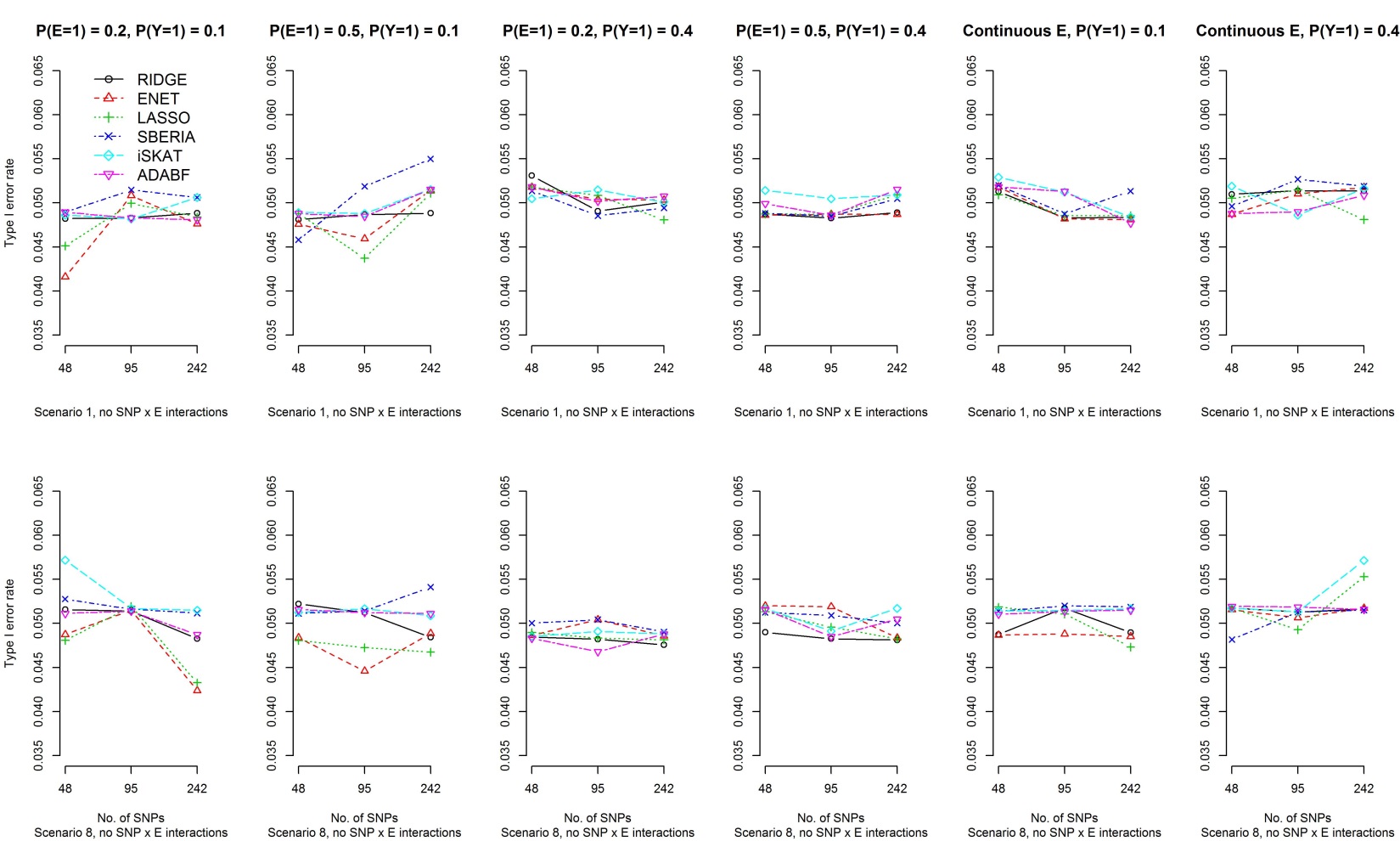


**Figure S1** Empirical type I error rates under the nominal significance level of 0.05 (binary trait)


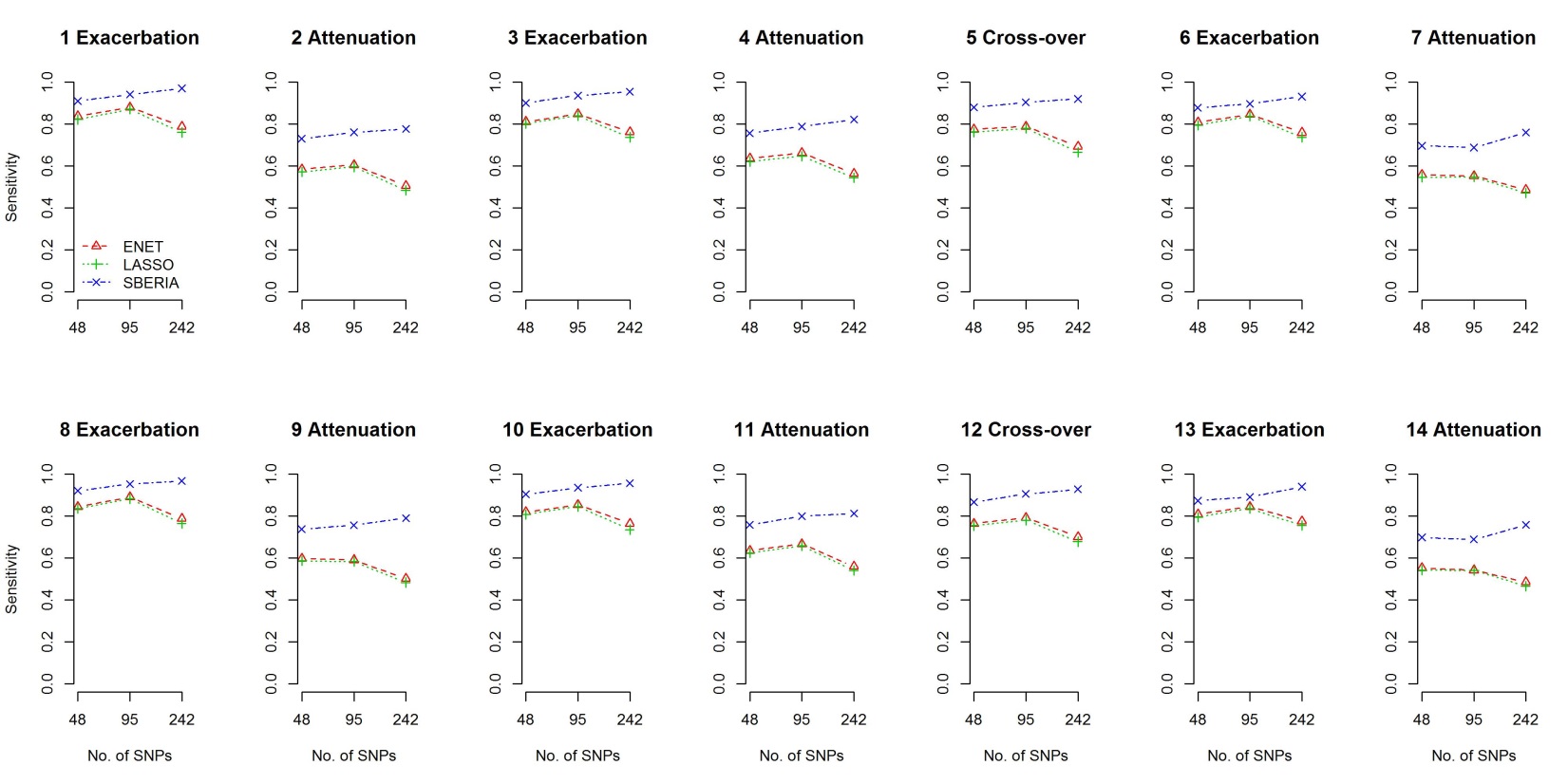


**Figure S2** The sensitivity of the marginal-association filtering in ENET, LASSO, and SBERIA, for continuous traits and $P\left( E=1 \right)=0.2$


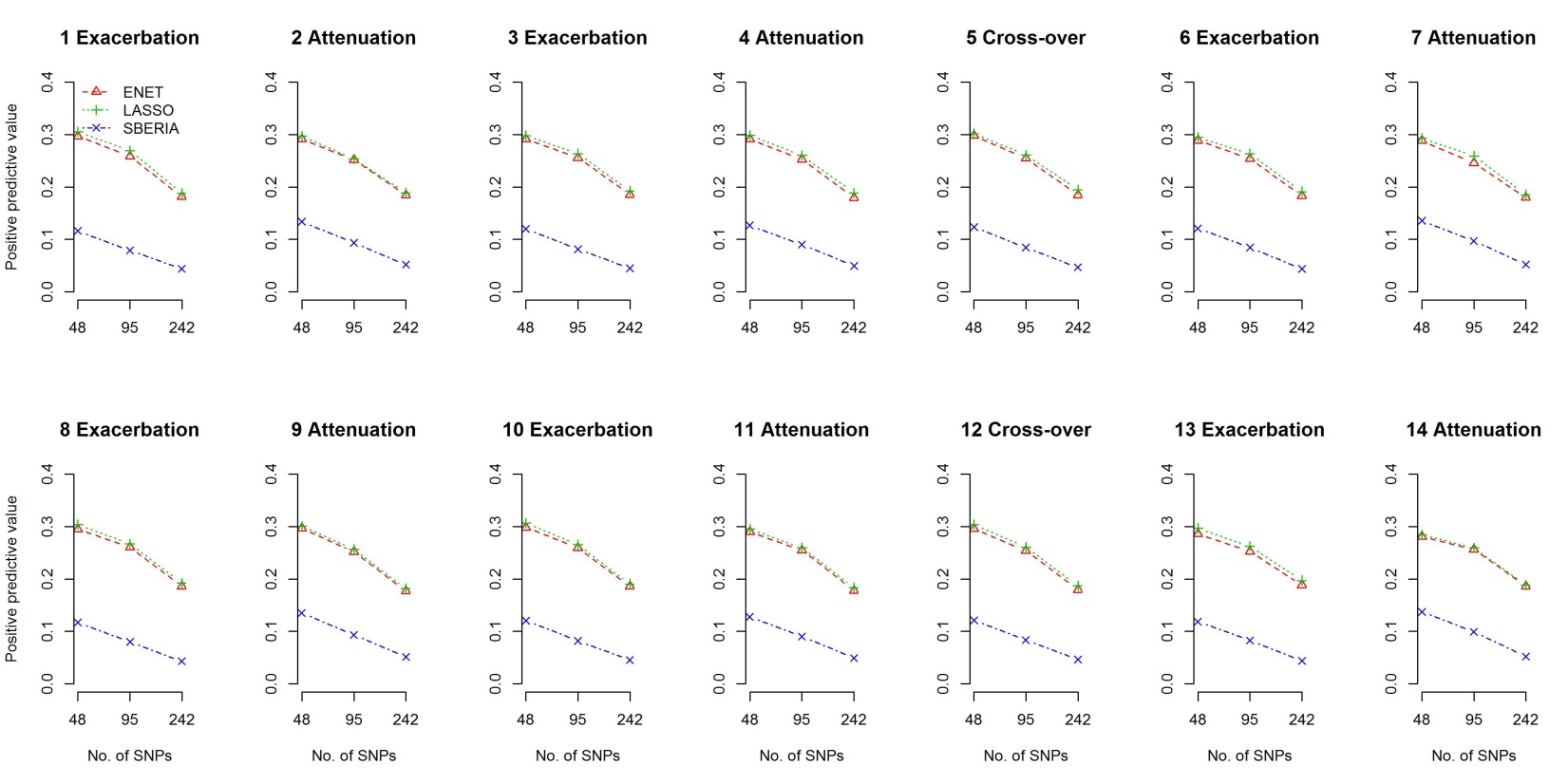


**Figure S3** The positive predictive value of the marginal-association filtering in ENET, LASSO, and SBERIA, for continuous traits and $P\left( E=1 \right)=0.2$


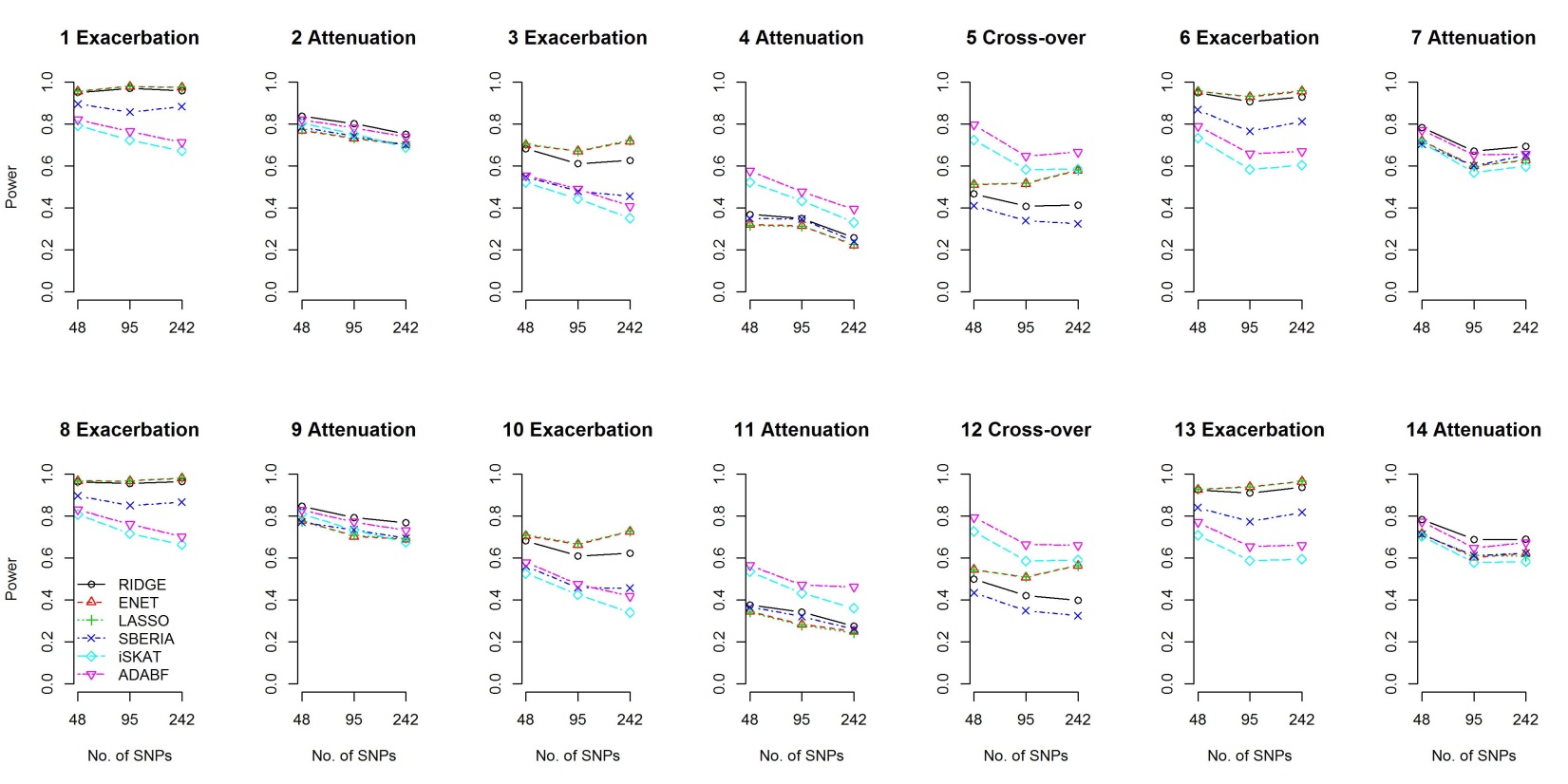


**Figure S4** Power given a significance level of 0.05, for continuous traits and $P\left( E=1 \right)=0.5$


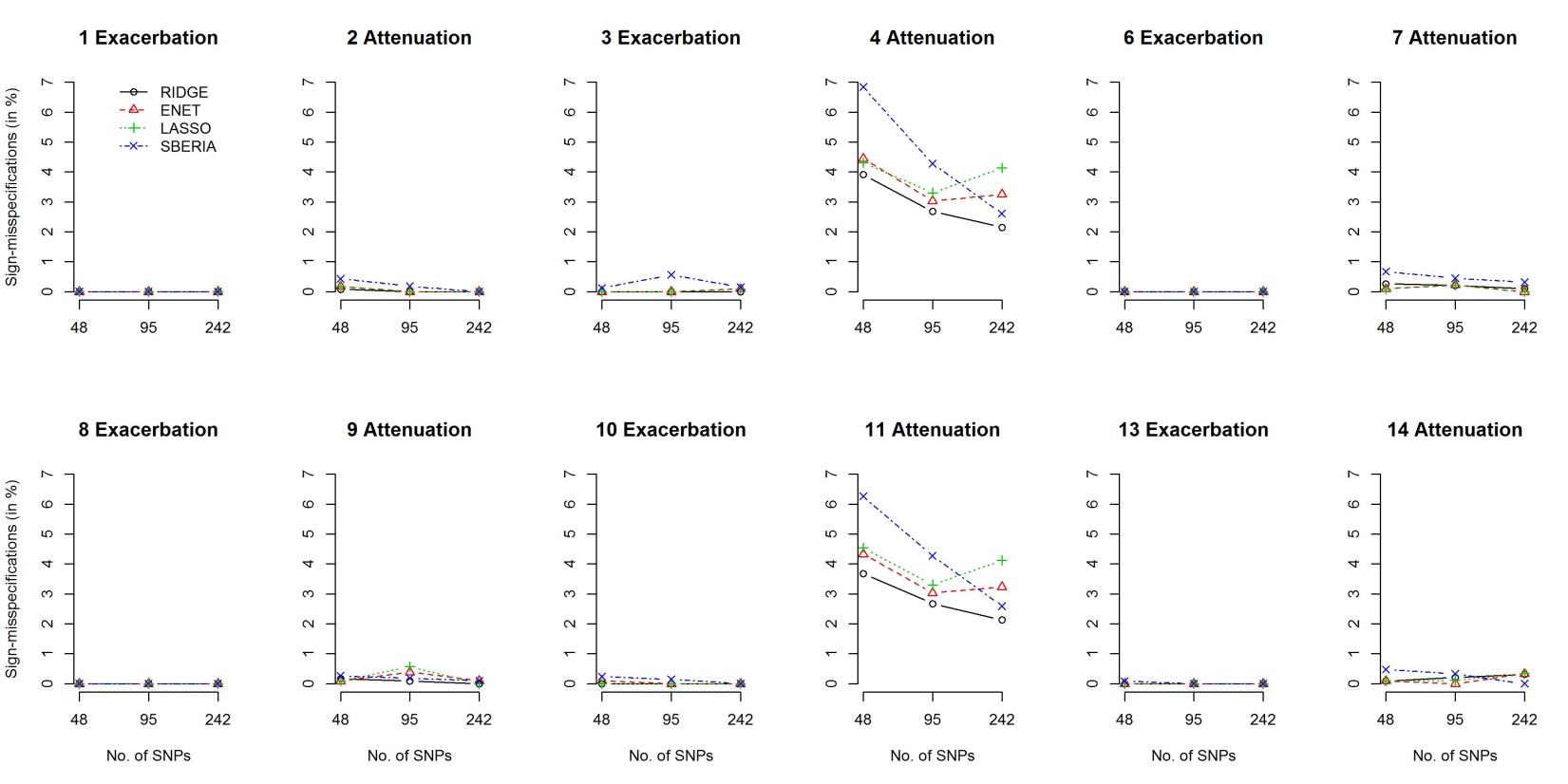


**Figure S5** Percentages of sign-misspecifications for $\gamma_{Int}$, under continuous traits and $P\left( E=1 \right)=0.5$


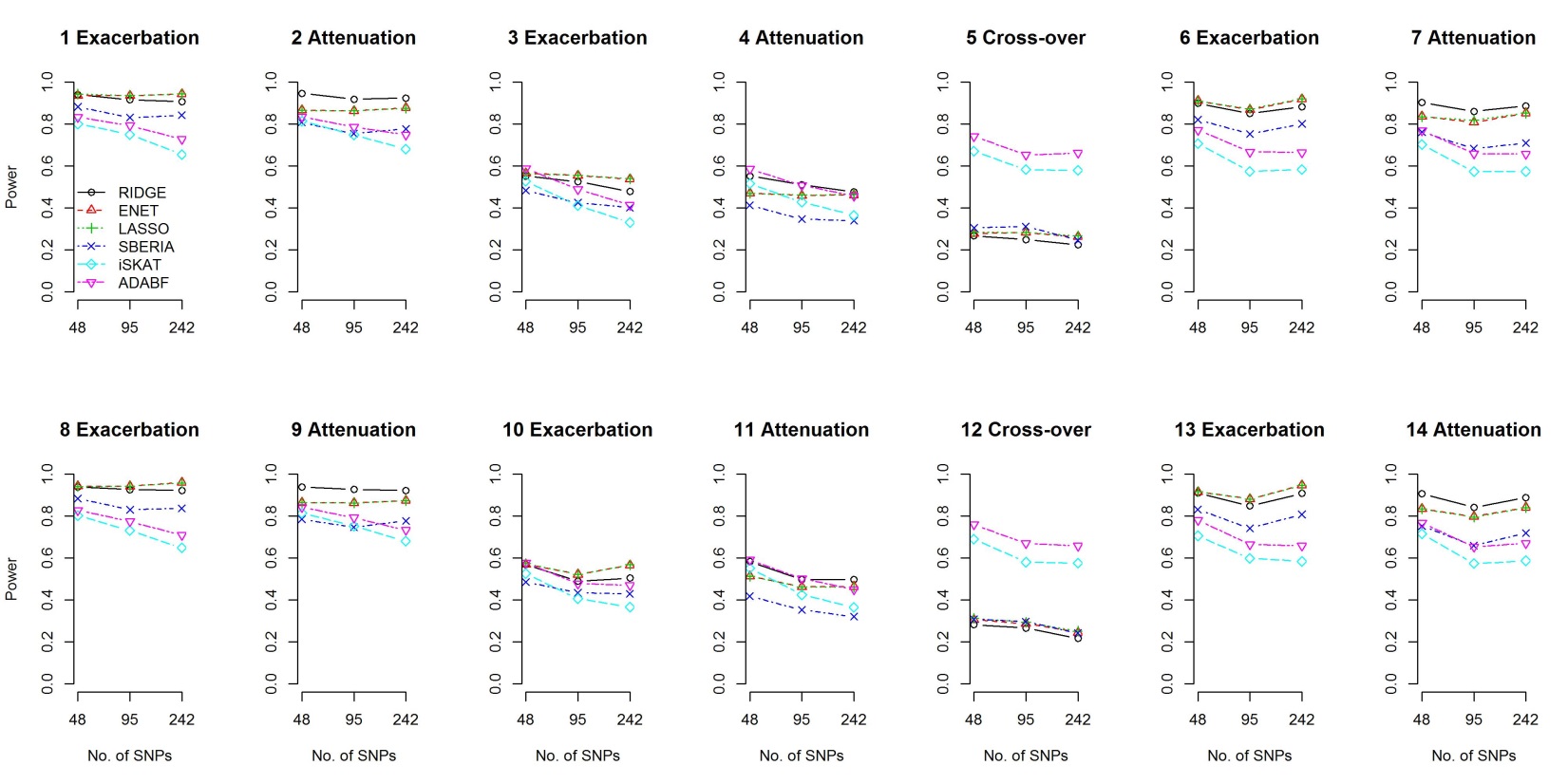


**Figure S6** Power given a significance level of 0.05, for continuous traits and a continuous *E*


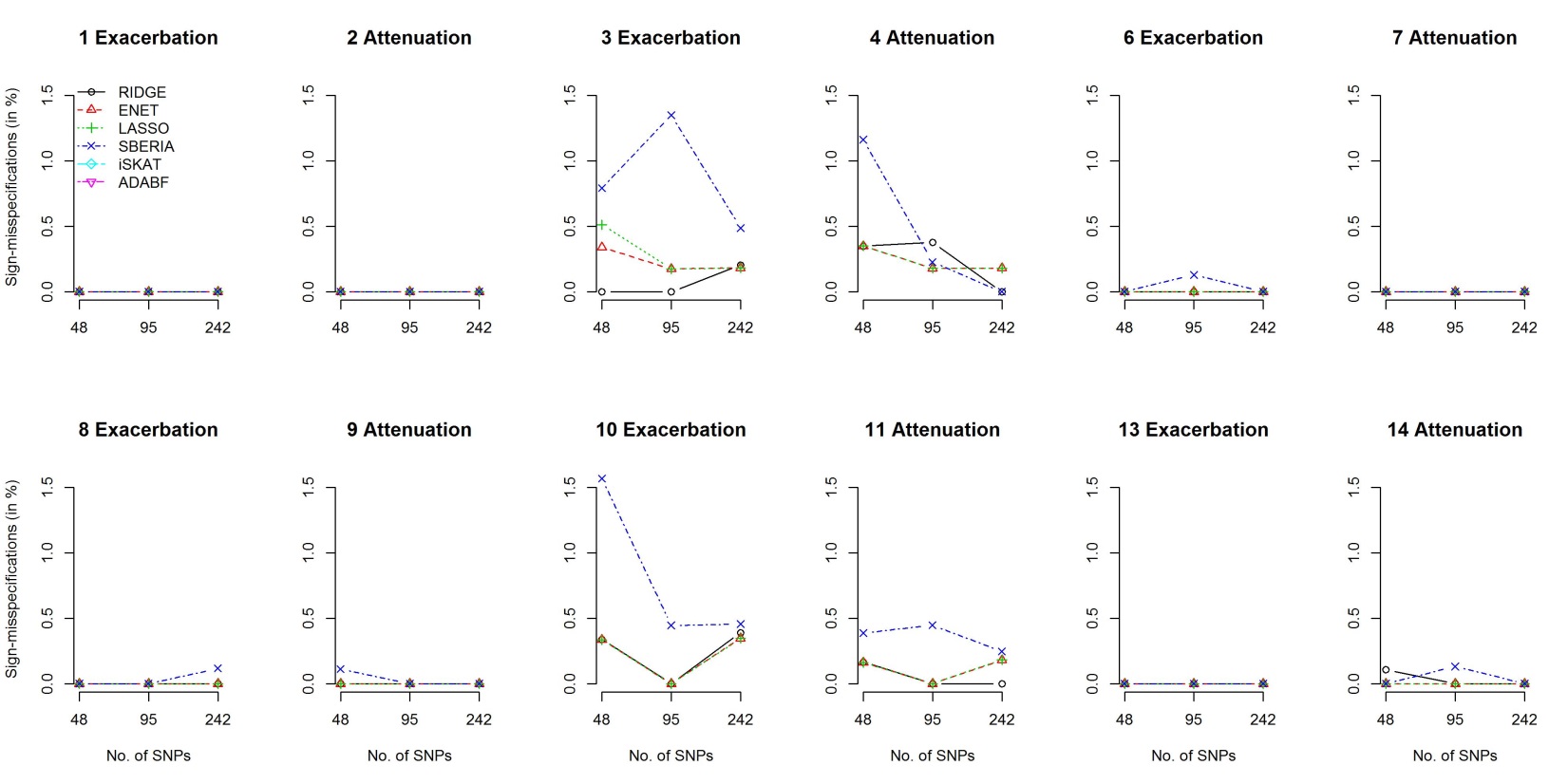


**Figure S7** Percentages of sign-misspecifications for $\gamma_{Int}$, under continuous traits and a continuous *E*


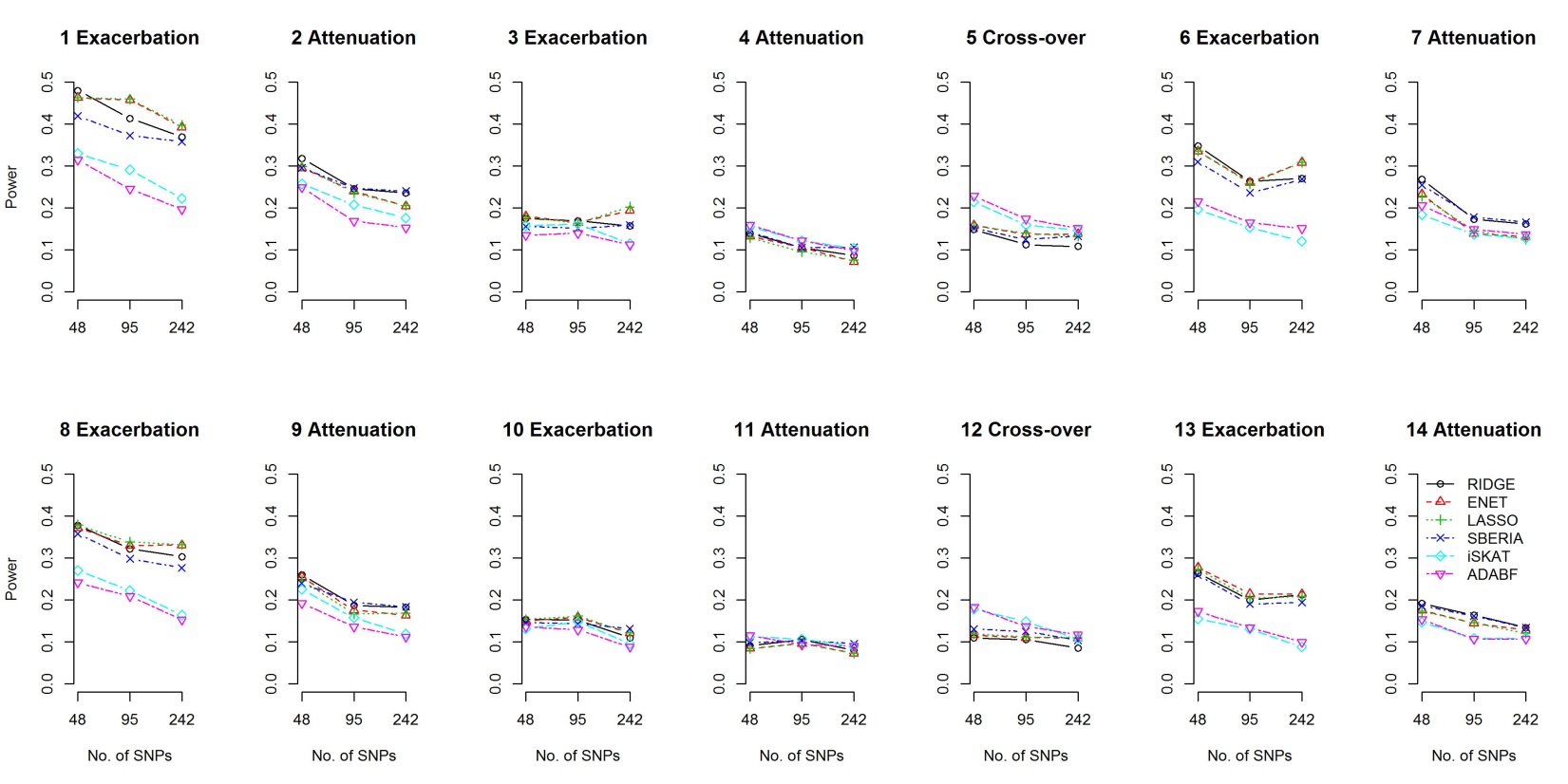


**Figure S8** Power given a significance level of 0.05, for binary traits, $P\left( Y=1 \right)=0.1$, and $P\left( E=1 \right)=0.2$


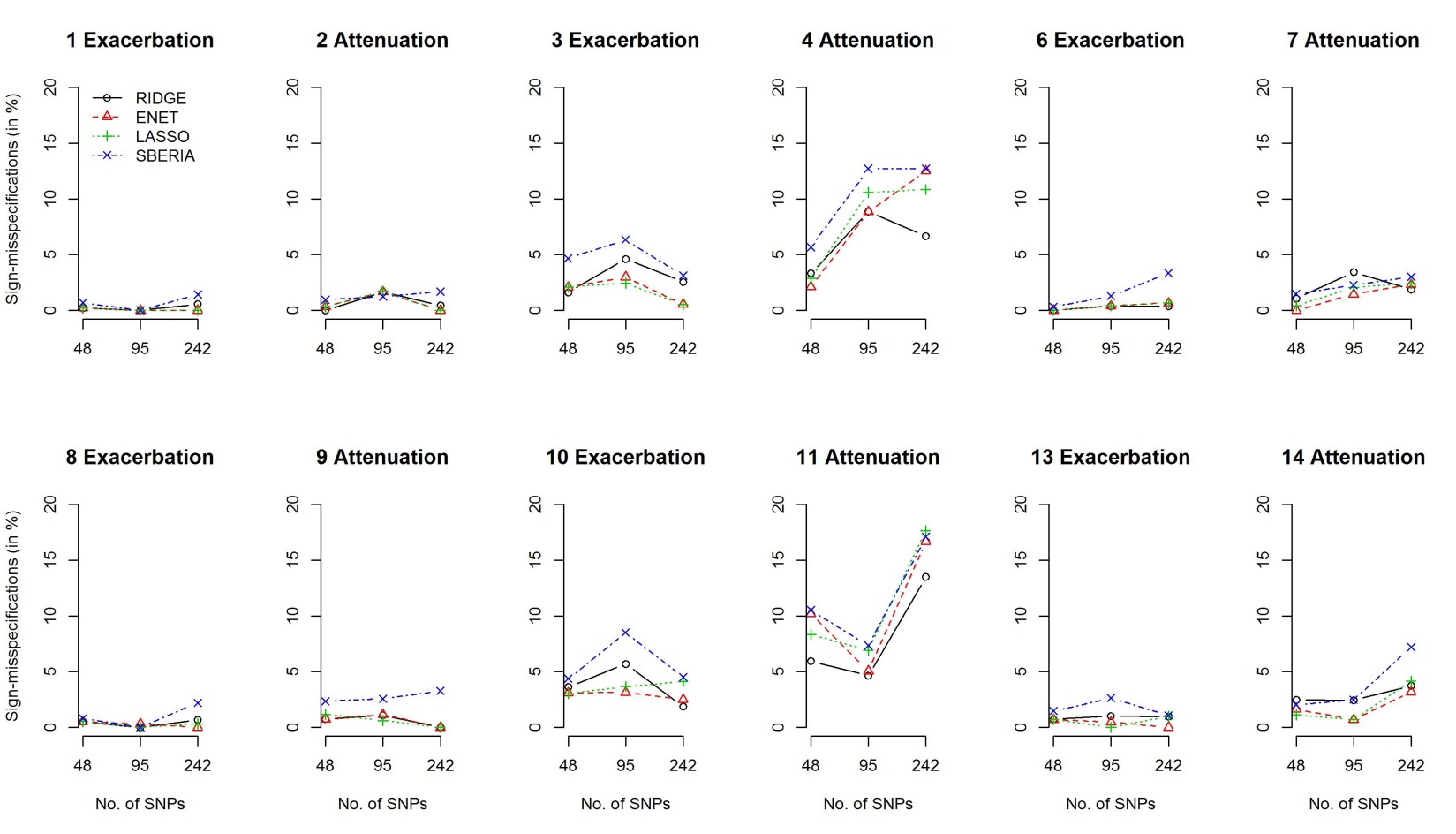


**Figure S9** Percentages of sign-misspecifications for $\gamma_{Int}$, under binary traits, $P\left( Y=1 \right)=0.1$, and $P\left( E=1 \right)=0.2$


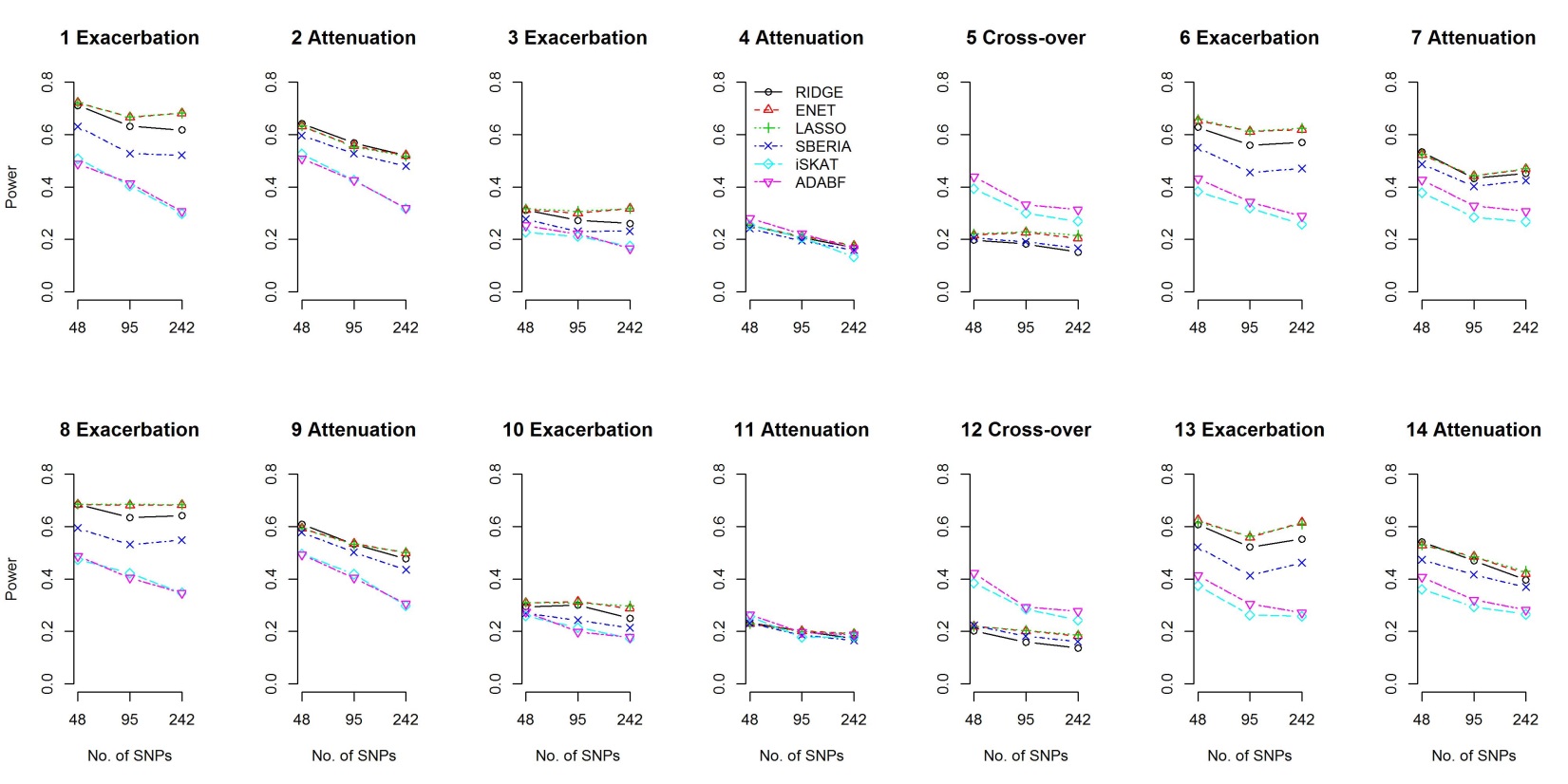


**Figure S10** Power given a significance level of 0.05, for binary traits, $P\left( Y=1 \right)=0.4$, and $P\left( E=1 \right)=0.2$


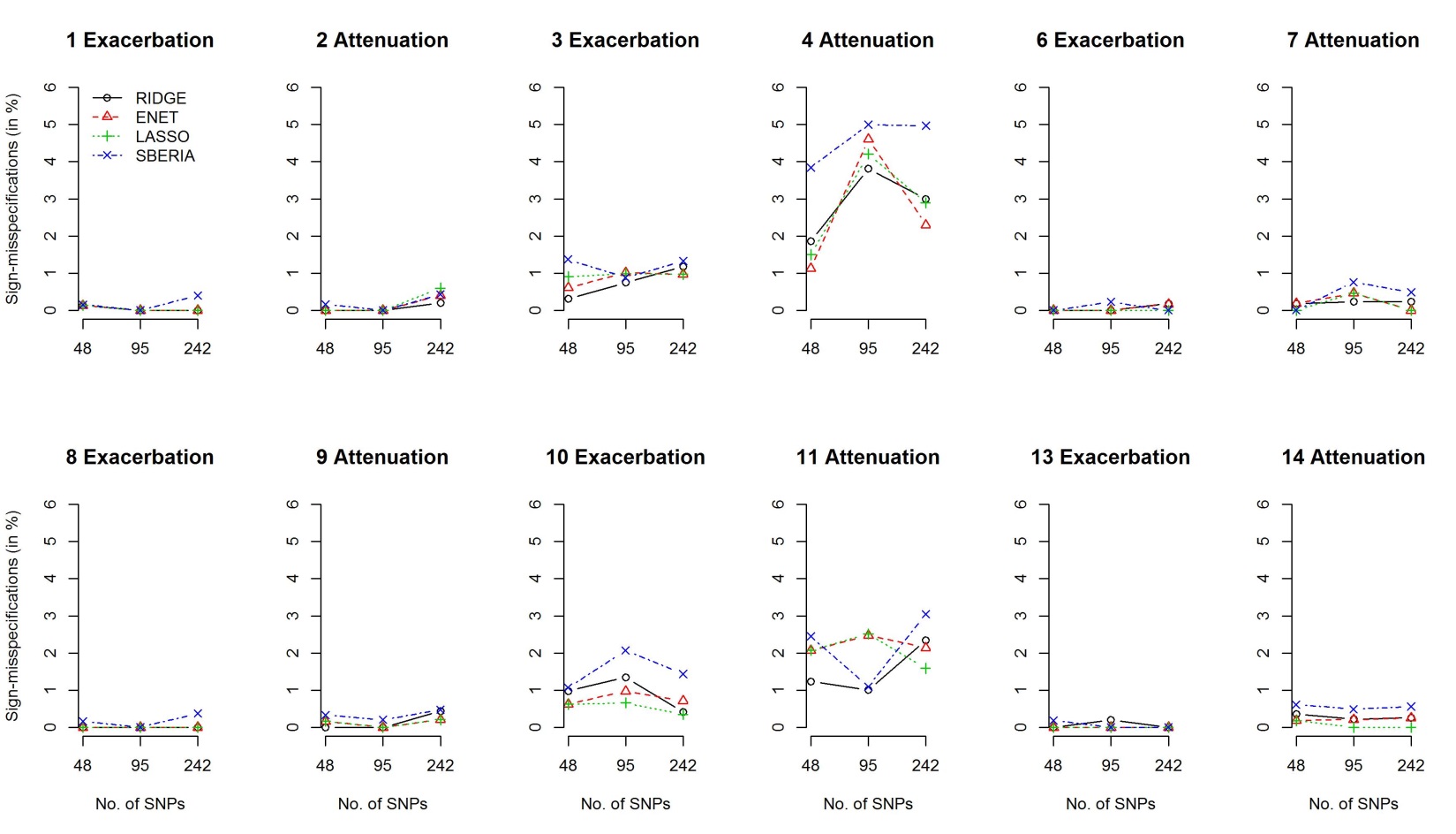


**Figure S11** Percentages of sign-misspecifications for $\gamma_{Int}$, under binary traits, $P\left( Y=1 \right)=0.4$, and $P\left( E=1 \right)=0.2$


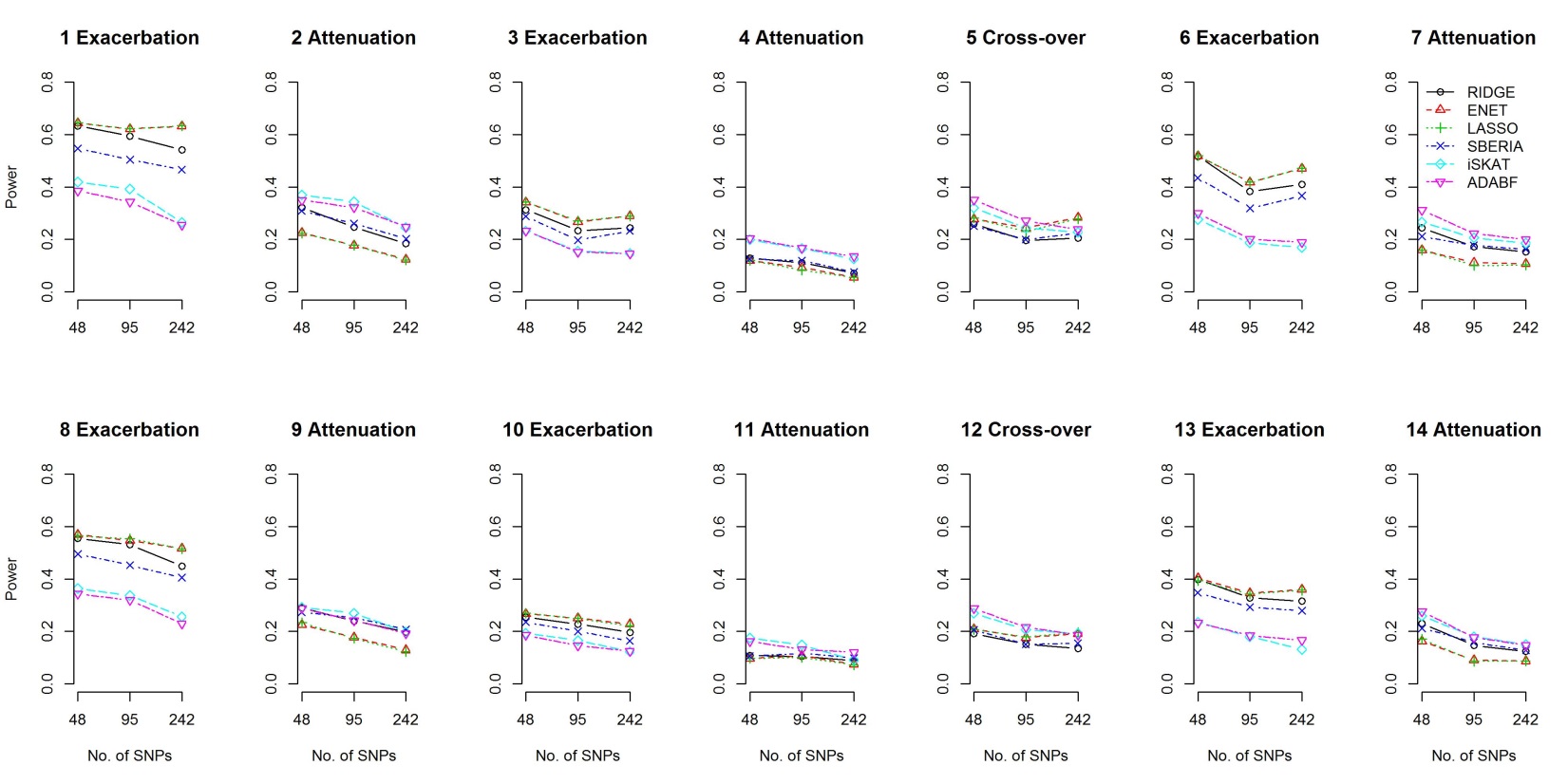


**Figure S12** Power given a significance level of 0.05, for binary traits, $P\left( Y=1 \right)=0.1$, and $P\left( E=1 \right)=0.5$


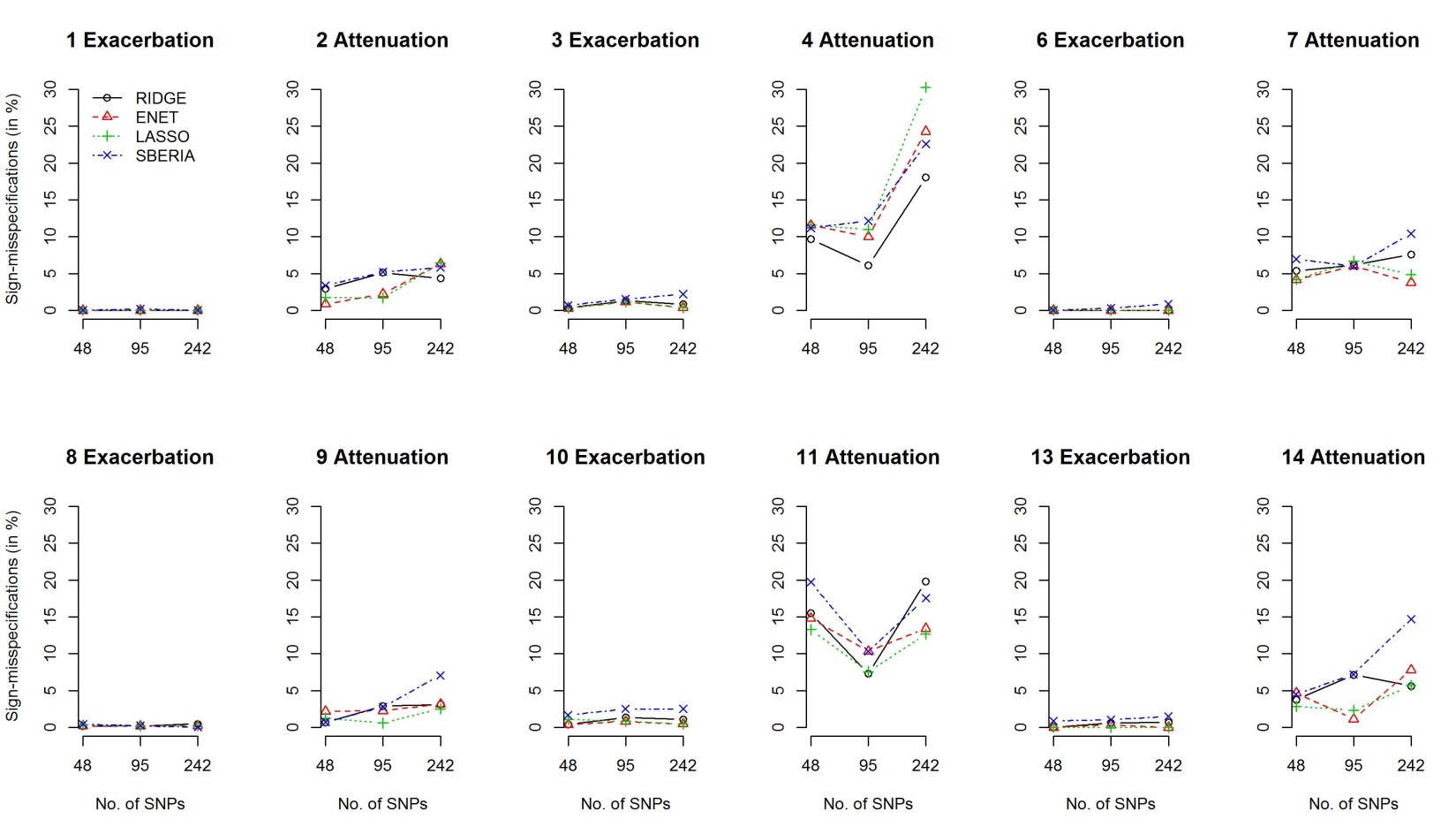


**Figure S13** Percentages of sign-misspecifications for $\gamma_{Int}$, under binary traits, $P\left( Y=1 \right)=0.1$, and $P\left( E=1 \right)=0.5$


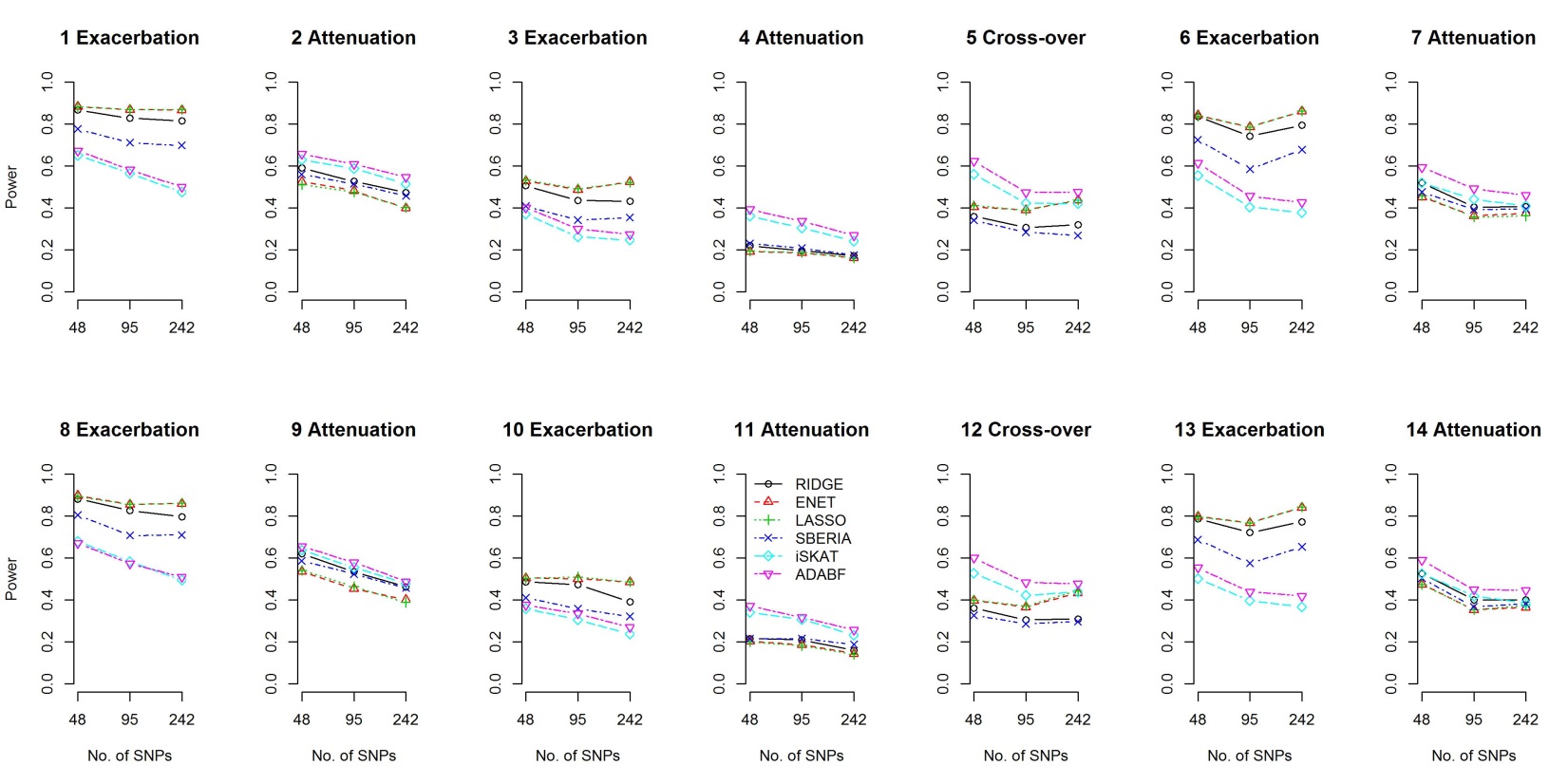


**Figure S14** Power given a significance level of 0.05, for binary traits, $P\left( Y=1 \right)=0.4$, and $P\left( E=1 \right)=0.5$


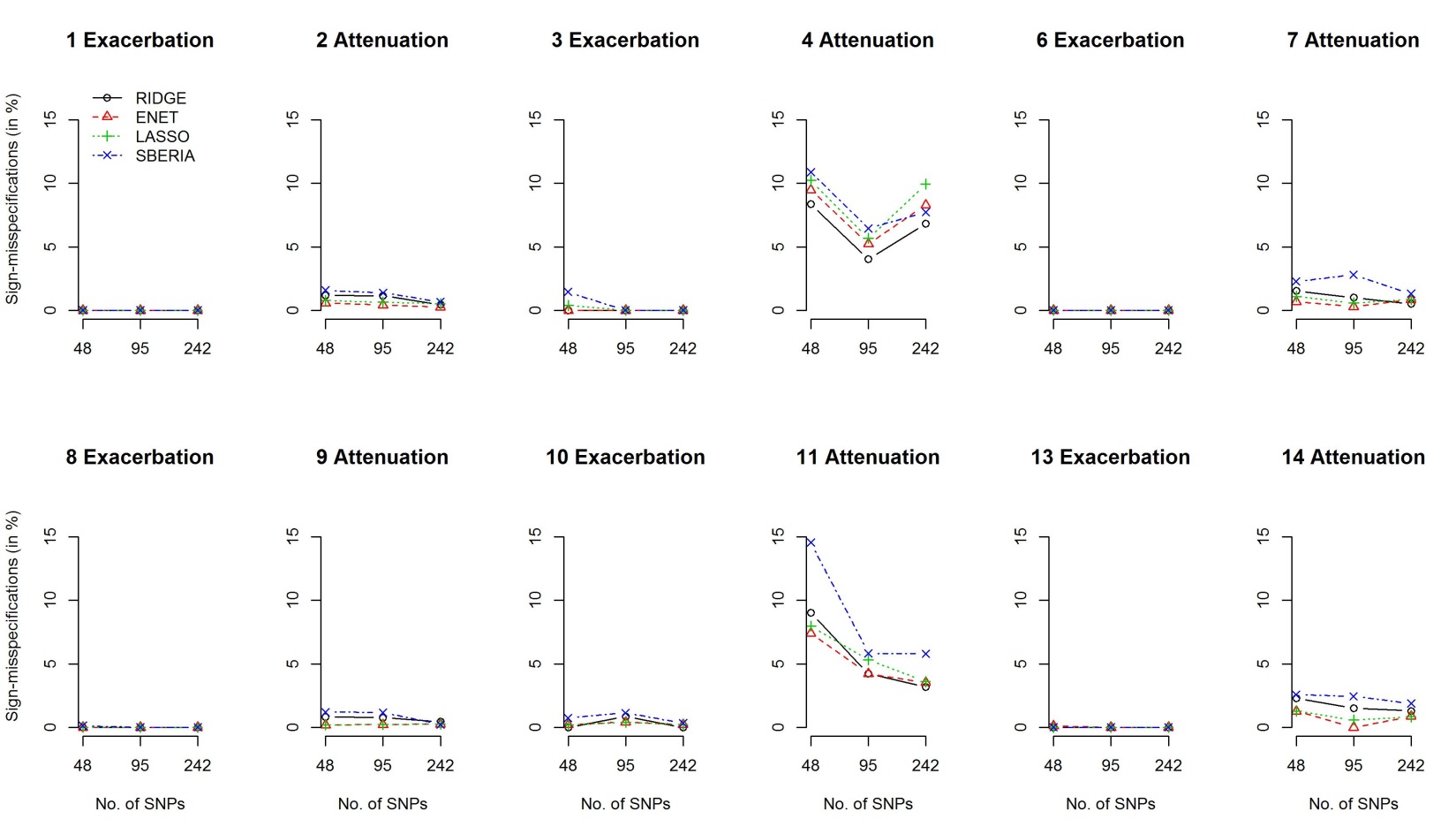


**Figure S15** Percentages of sign-misspecifications for $\gamma_{Int}$, under binary traits, $P\left( Y=1 \right)=0.4$, and $P\left( E=1 \right)=0.5$


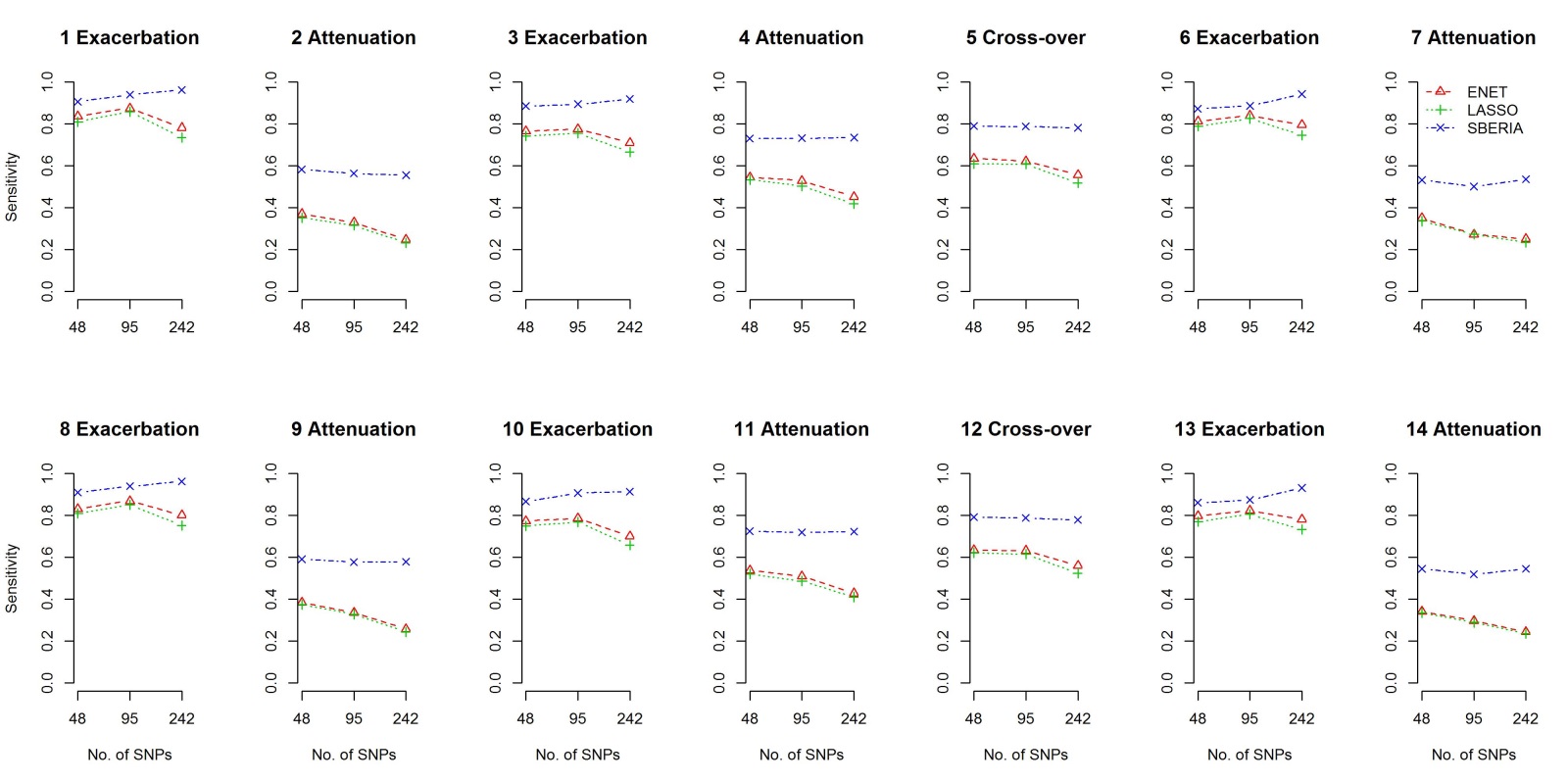


**Figure S16** The sensitivity of the marginal-association filtering in ENET, LASSO, and SBERIA, for binary traits, $P\left( Y=1 \right)=0.4$, and $P\left( E=1 \right)=0.5$


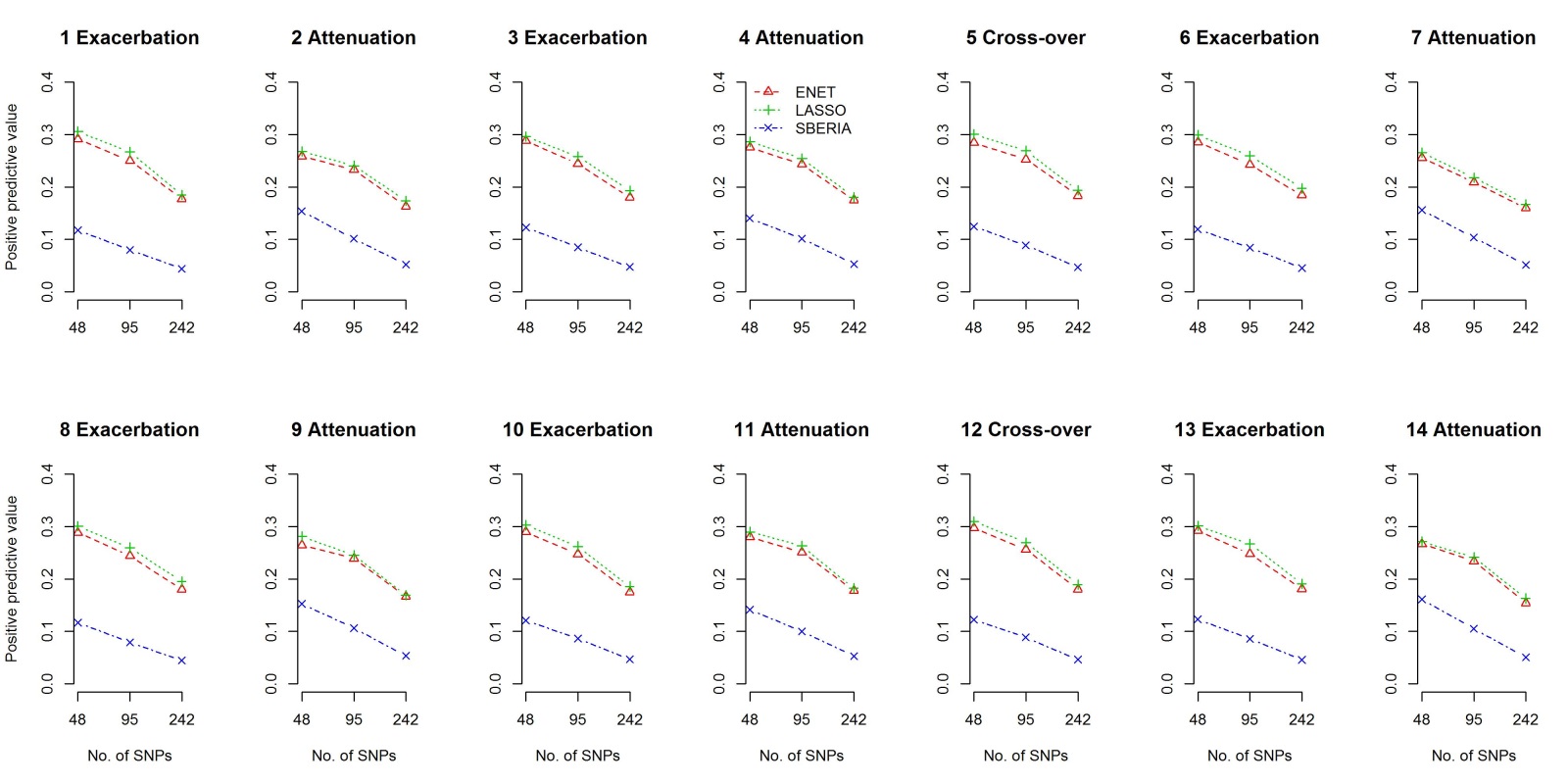


**Figure S17** The positive predictive value of the marginal-association filtering in ENET, LASSO, and SBERIA, for binary traits, $P\left( Y=1 \right)=0.4$, and $P\left( E=1 \right)=0.5$

**
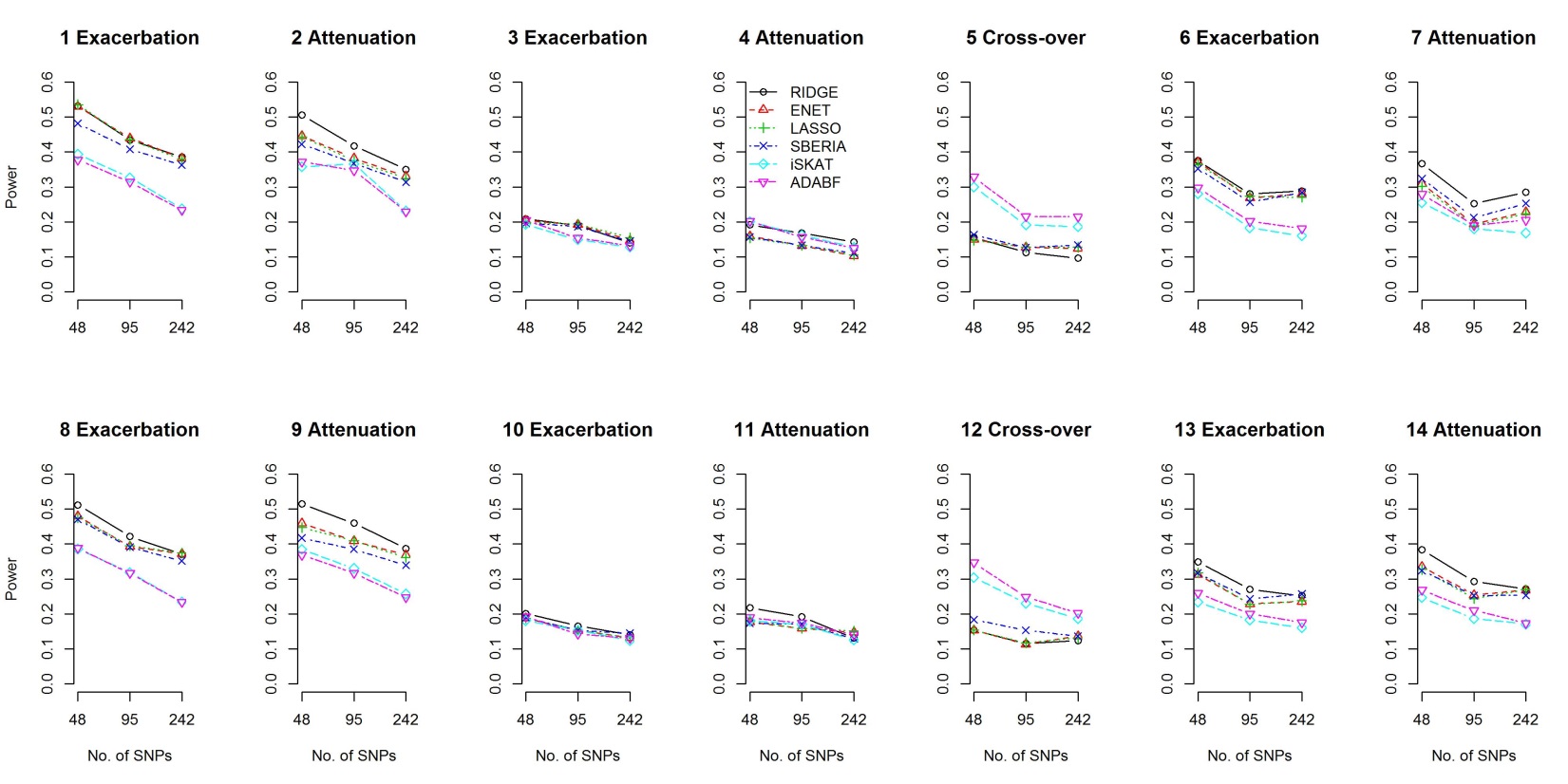
**

**Figure S18** Power given a significance level of 0.05, for binary traits, $P\left( Y=1 \right)=0.1$, and a continuous E


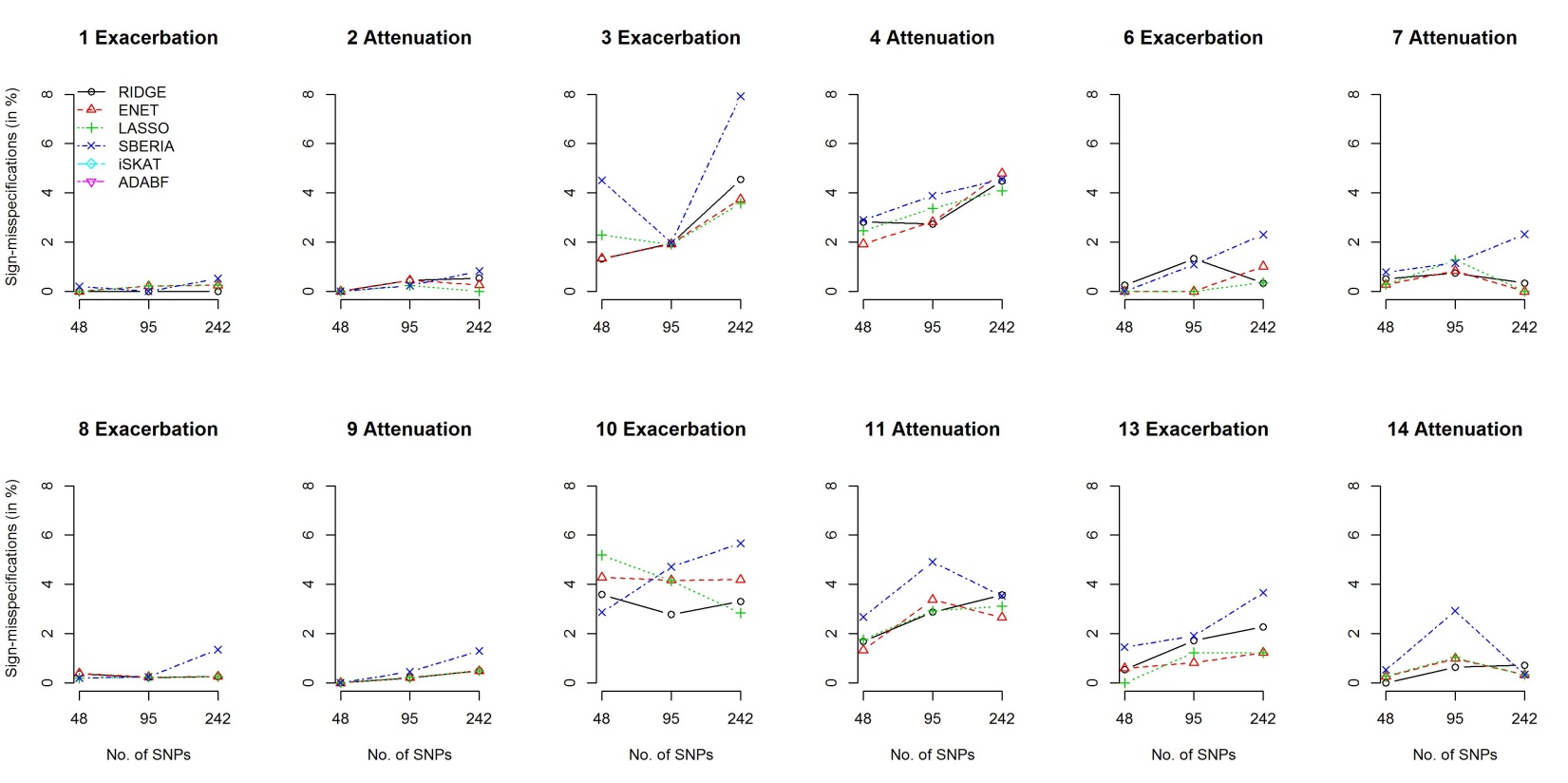


**Figure S19** Percentages of sign-misspecifications for $\gamma_{Int}$, under binary traits, $P\left( Y=1 \right)=0.1$, and a continuous E


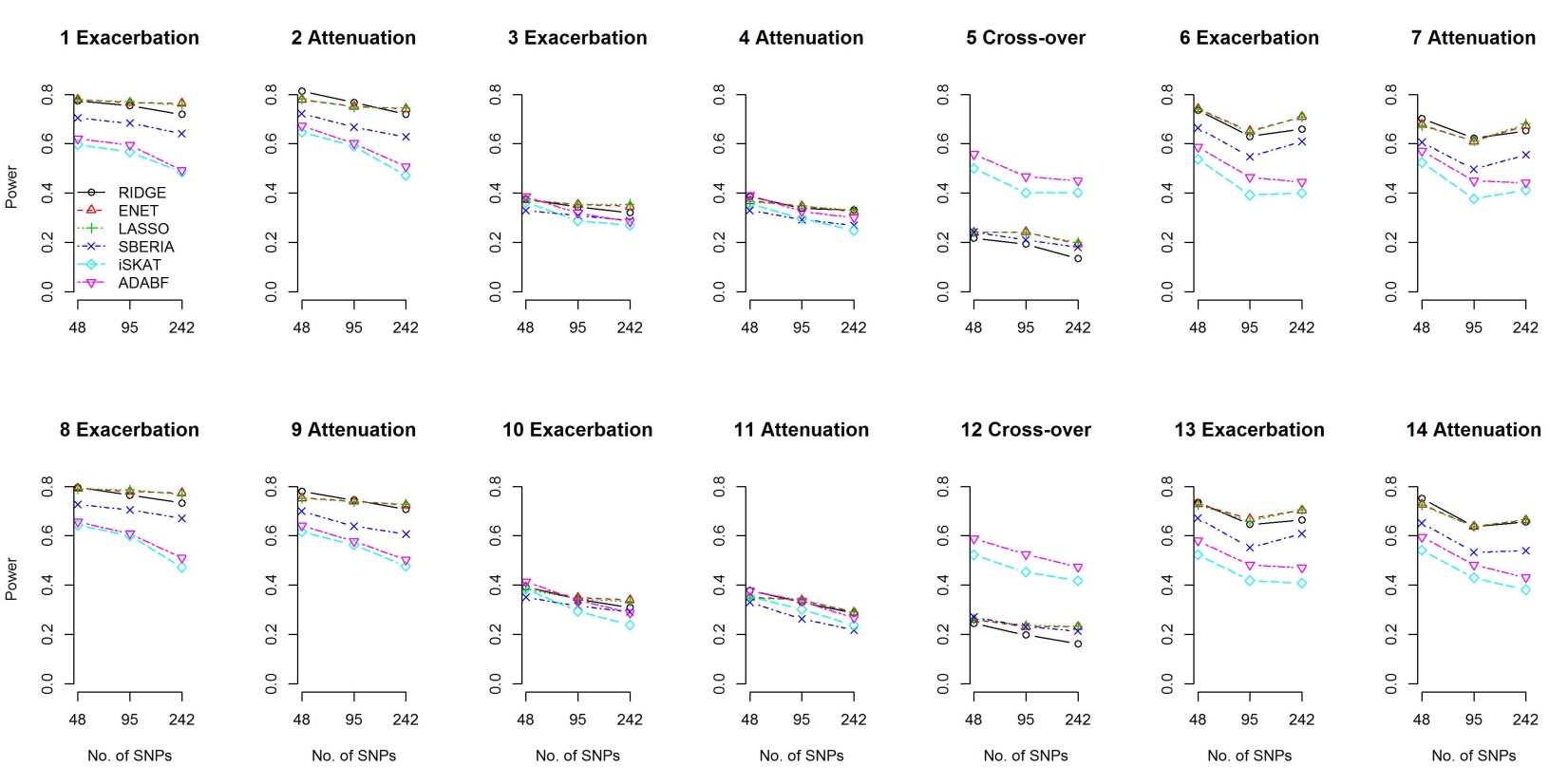


**Figure S20** Power given a significance level of 0.05, for binary traits, $P\left( Y=1 \right)=0.4$, and a continuous E


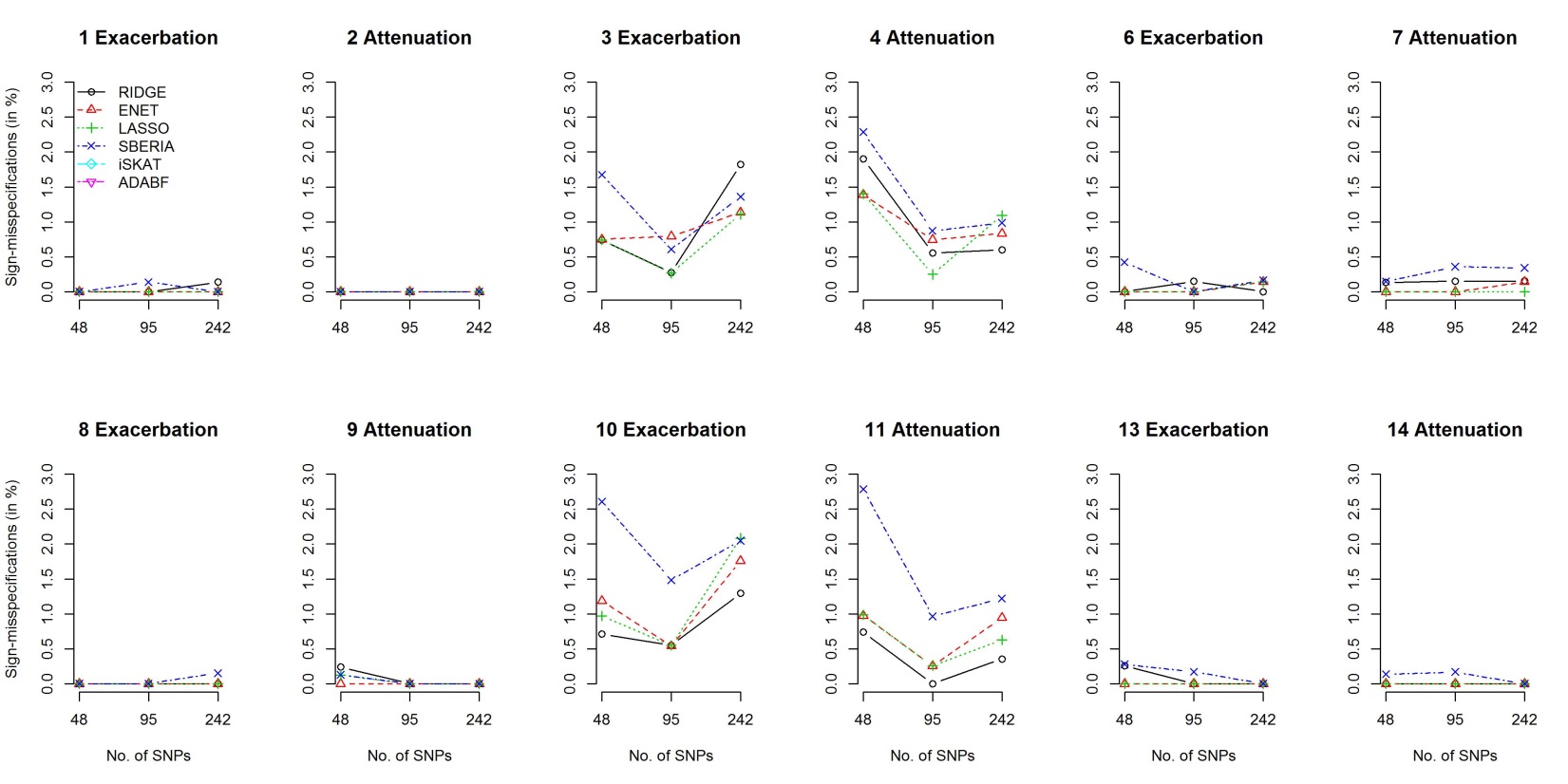


**Figure S21** Percentages of sign-misspecifications for $\gamma_{Int}$, under binary traits, $P\left( Y=1 \right)=0.4$, and a continuous E

**
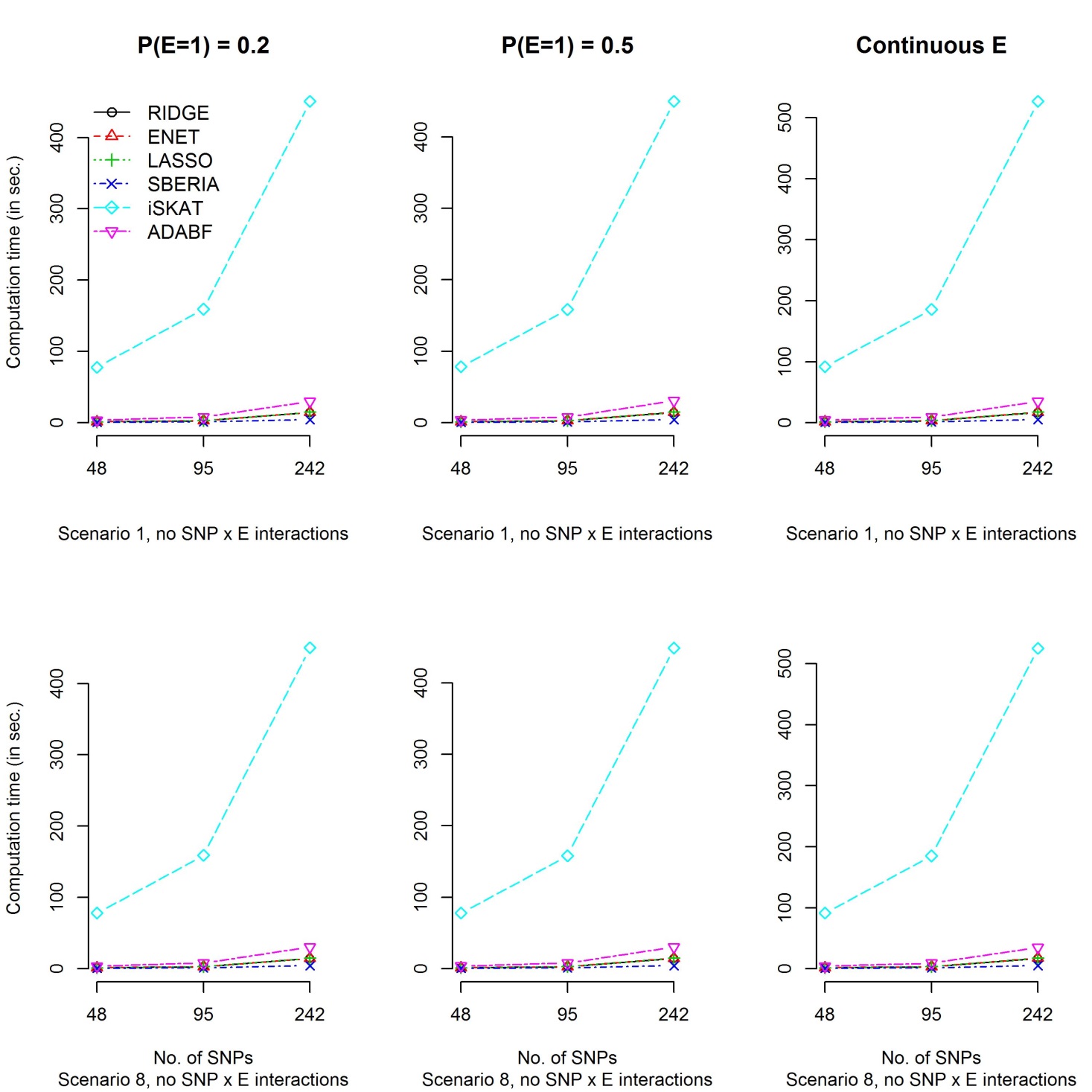
**

**Figure S22** Average time spent (in seconds) for each simulation replication, under $H_{0}$, for continuous traits.

**
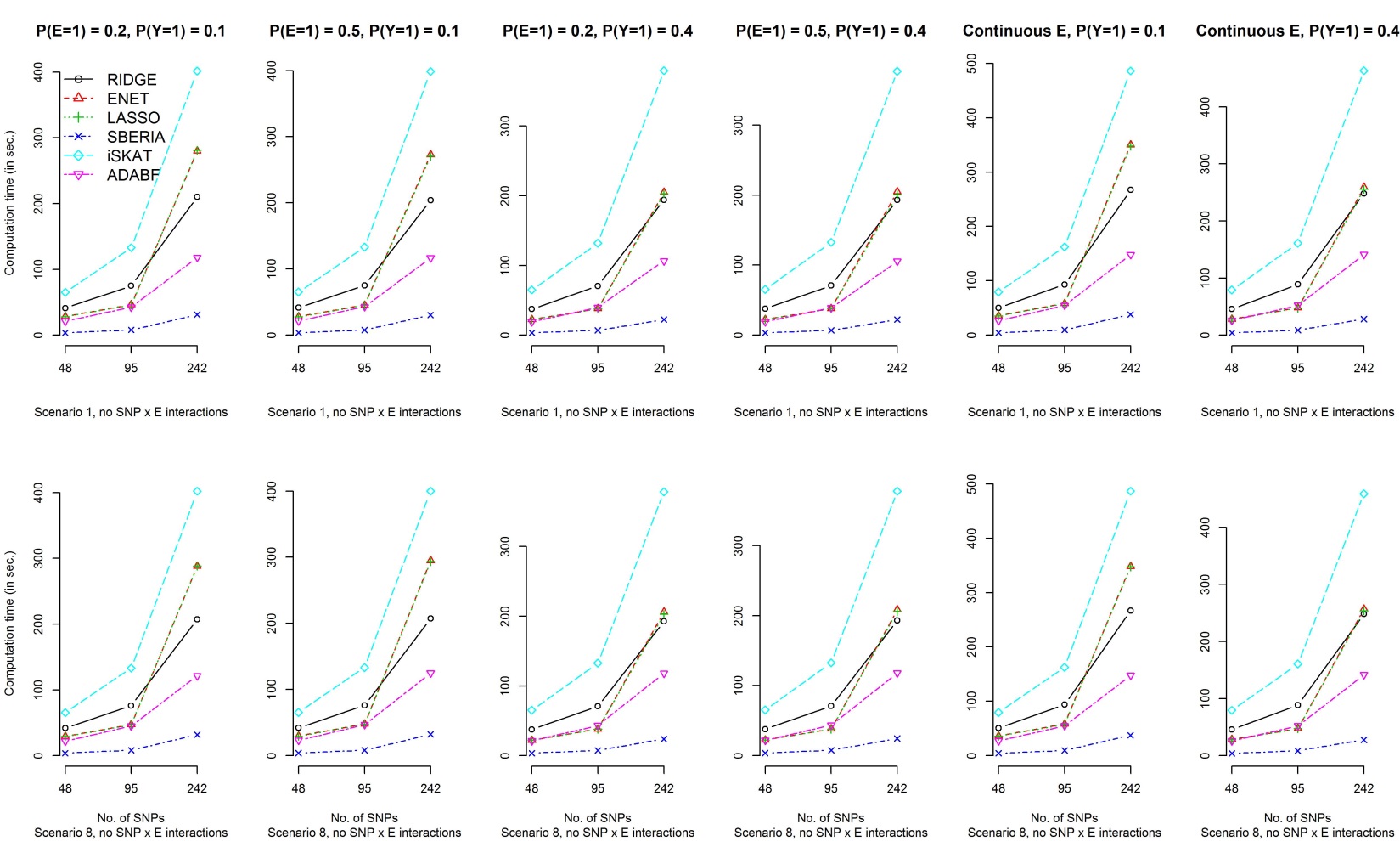
**

**Figure S23** Average time spent (in seconds) for each simulation replication, under $H_{0}$, for binary traits.


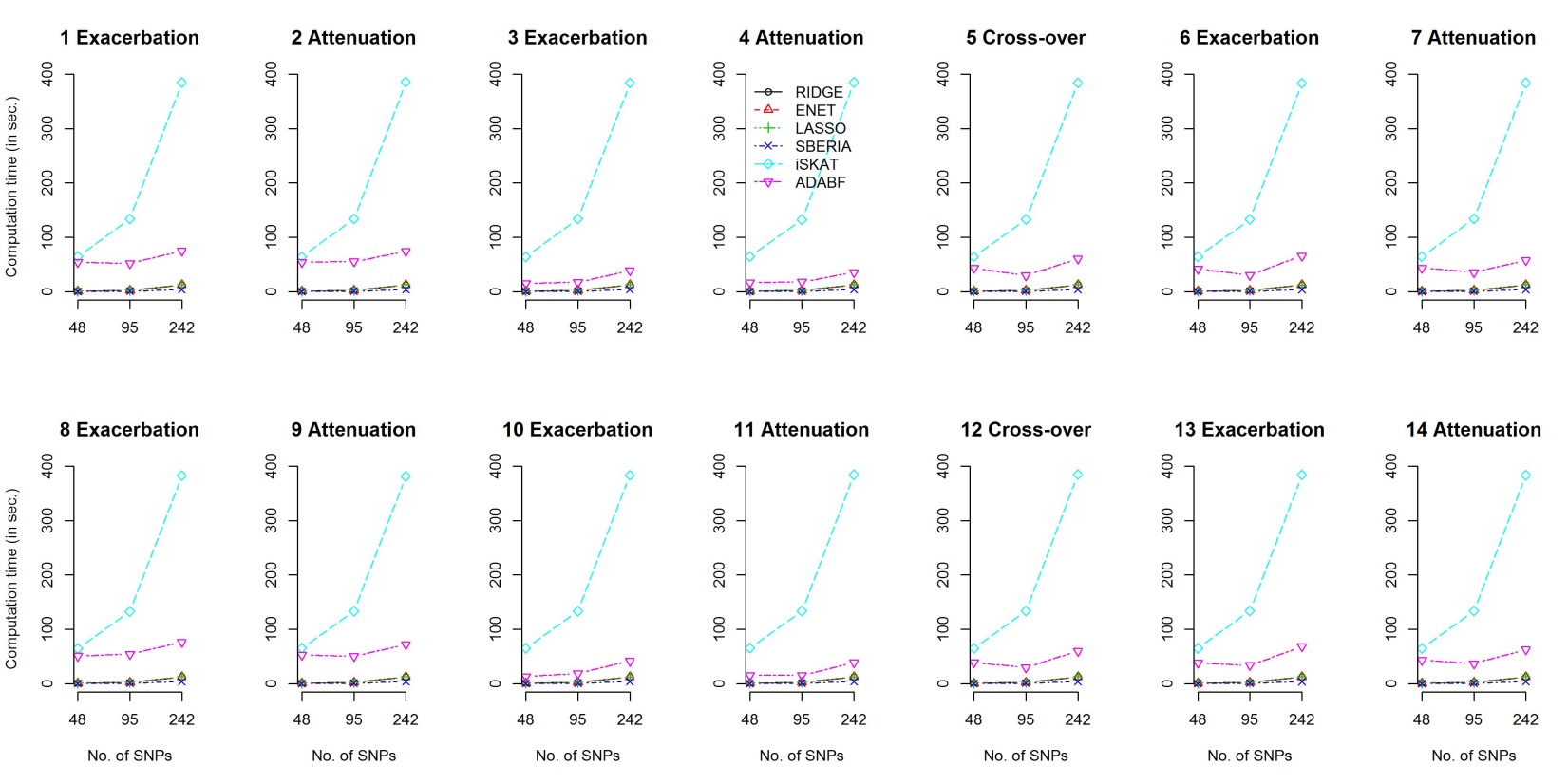


**Figure S24** Average time spent (in seconds) for each simulation replication, under $H_{1}$, for continuous traits and $P\left( E=1 \right)=0.2$


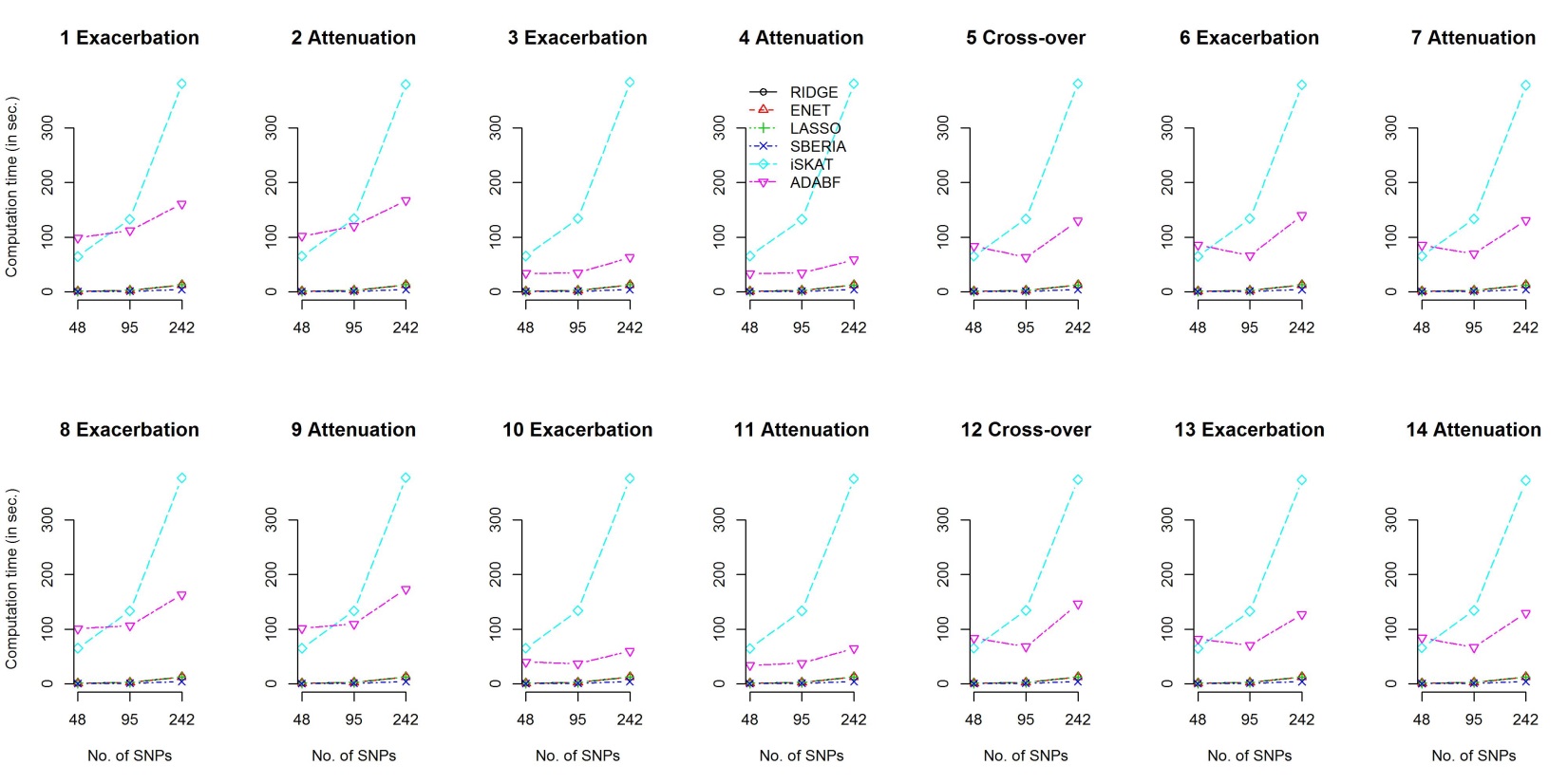


**Figure S25** Average time spent (in seconds) for each simulation replication, under $H_{1}$, for continuous traits and $P\left( E=1 \right)=0.5$


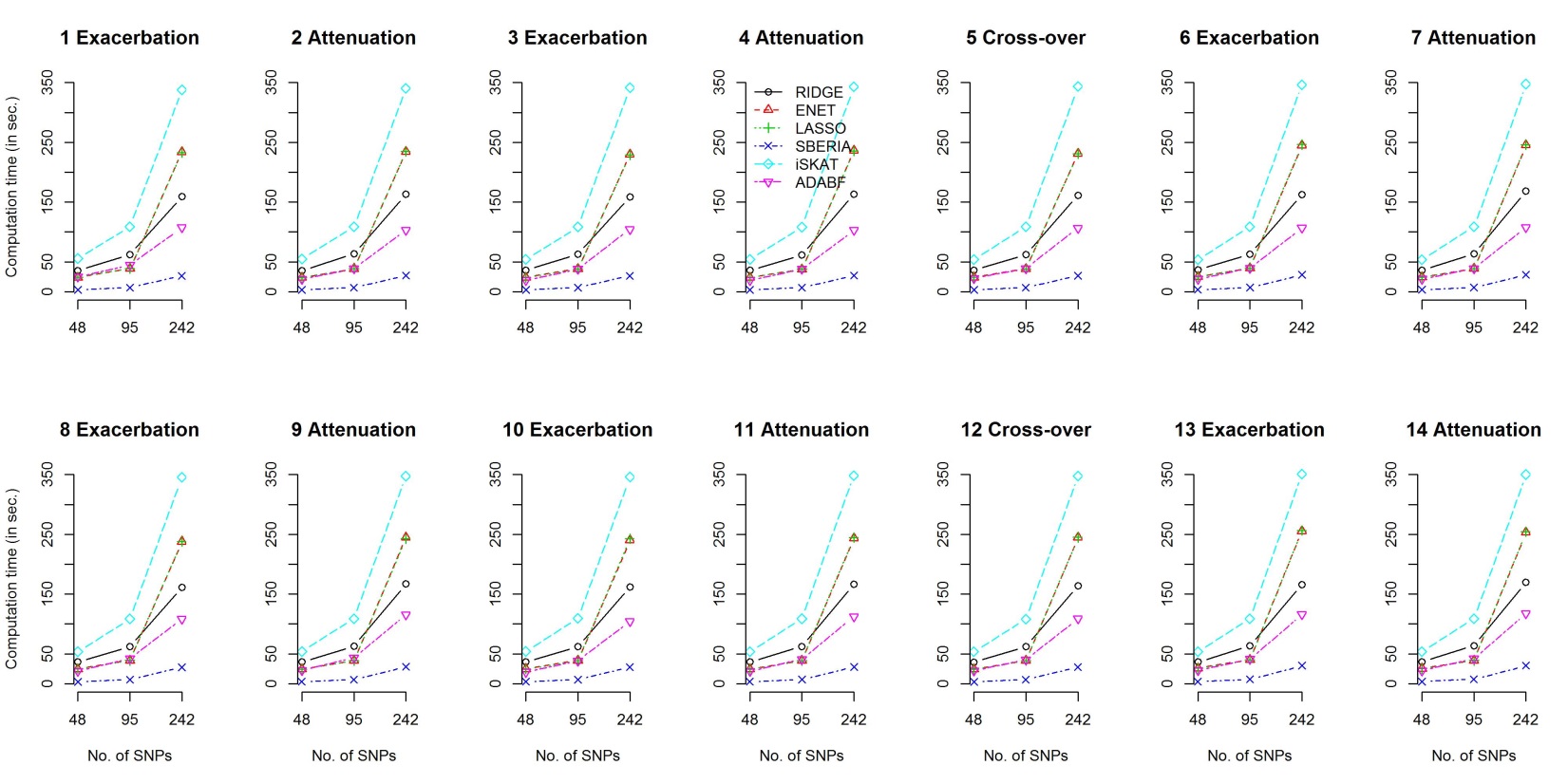


**Figure S26** Average time spent (in seconds) for each simulation replication, under $H_{1}$, for binary traits, $P\left( Y=1 \right)=0.1$, and $P\left( E=1 \right)=0.2$


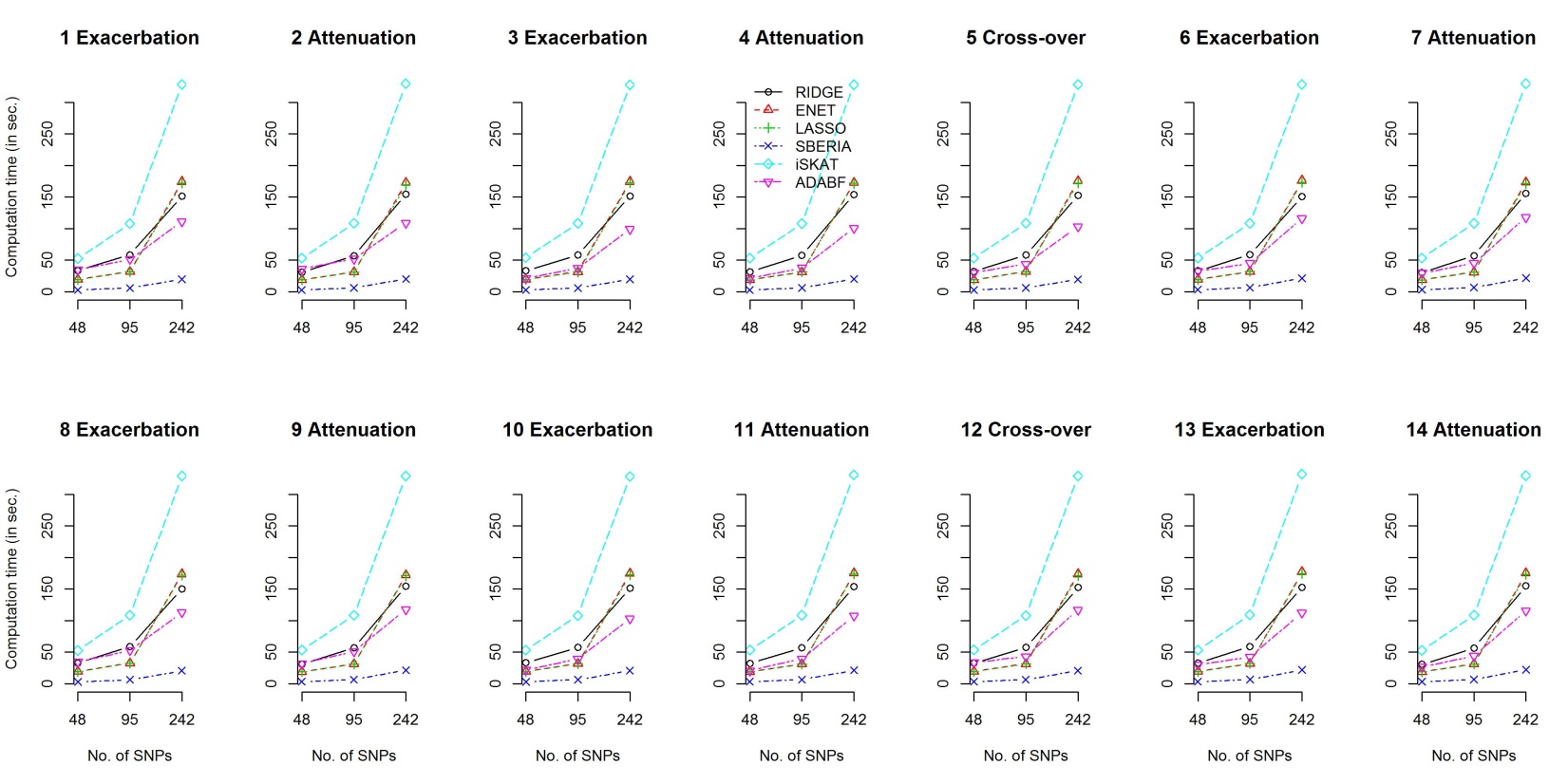


**Figure S27** Average time spent (in seconds) for each simulation replication, under $H_{1}$, for binary traits, $P\left( Y=1 \right)=0.4$, and $P\left( E=1 \right)=0.2$


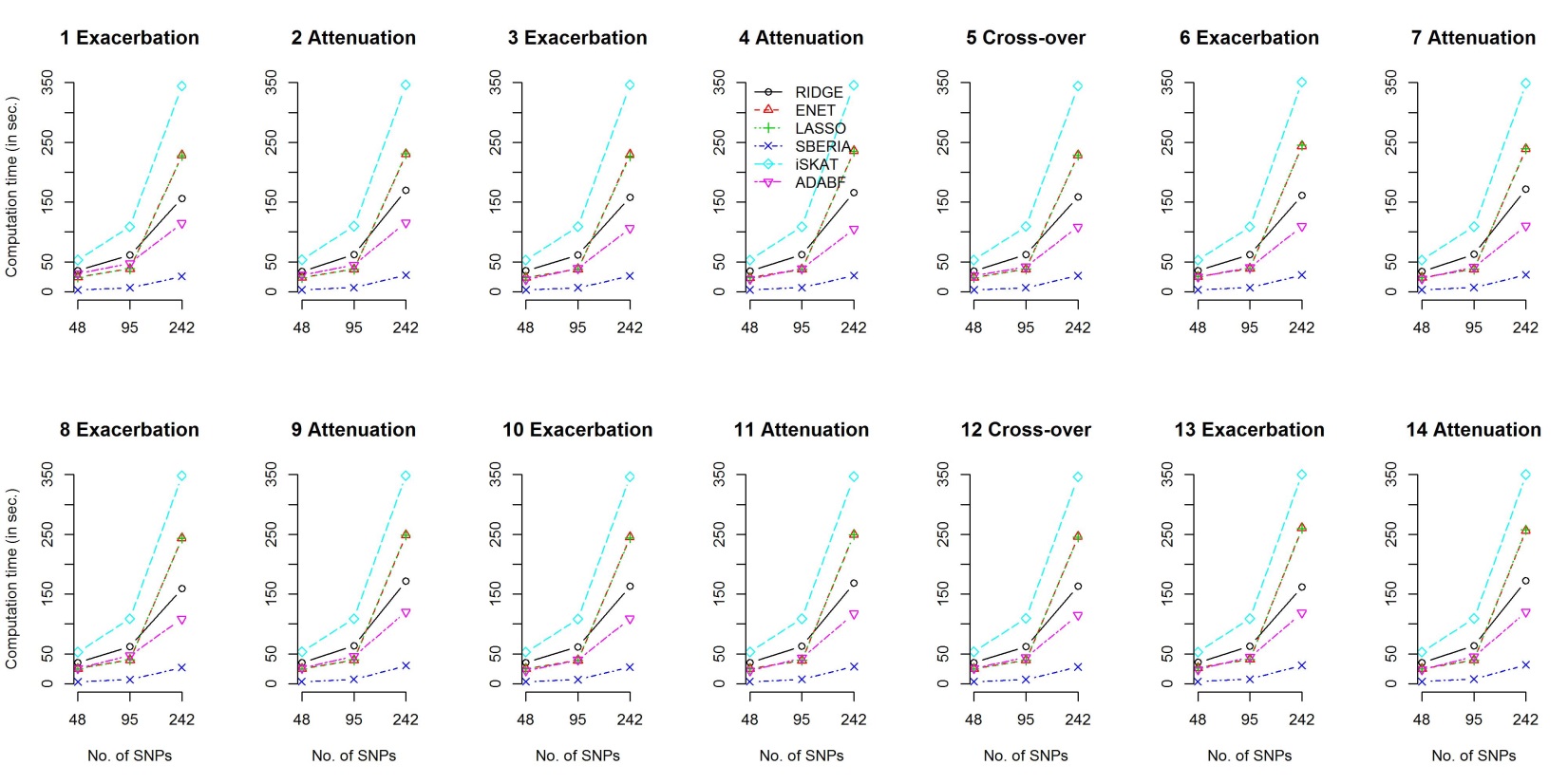


**Figure S28** Average time spent (in seconds) for each simulation replication, under $H_{1}$, for binary traits, $P\left( Y=1 \right)=0.1$, and $P\left( E=1 \right)=0.5$


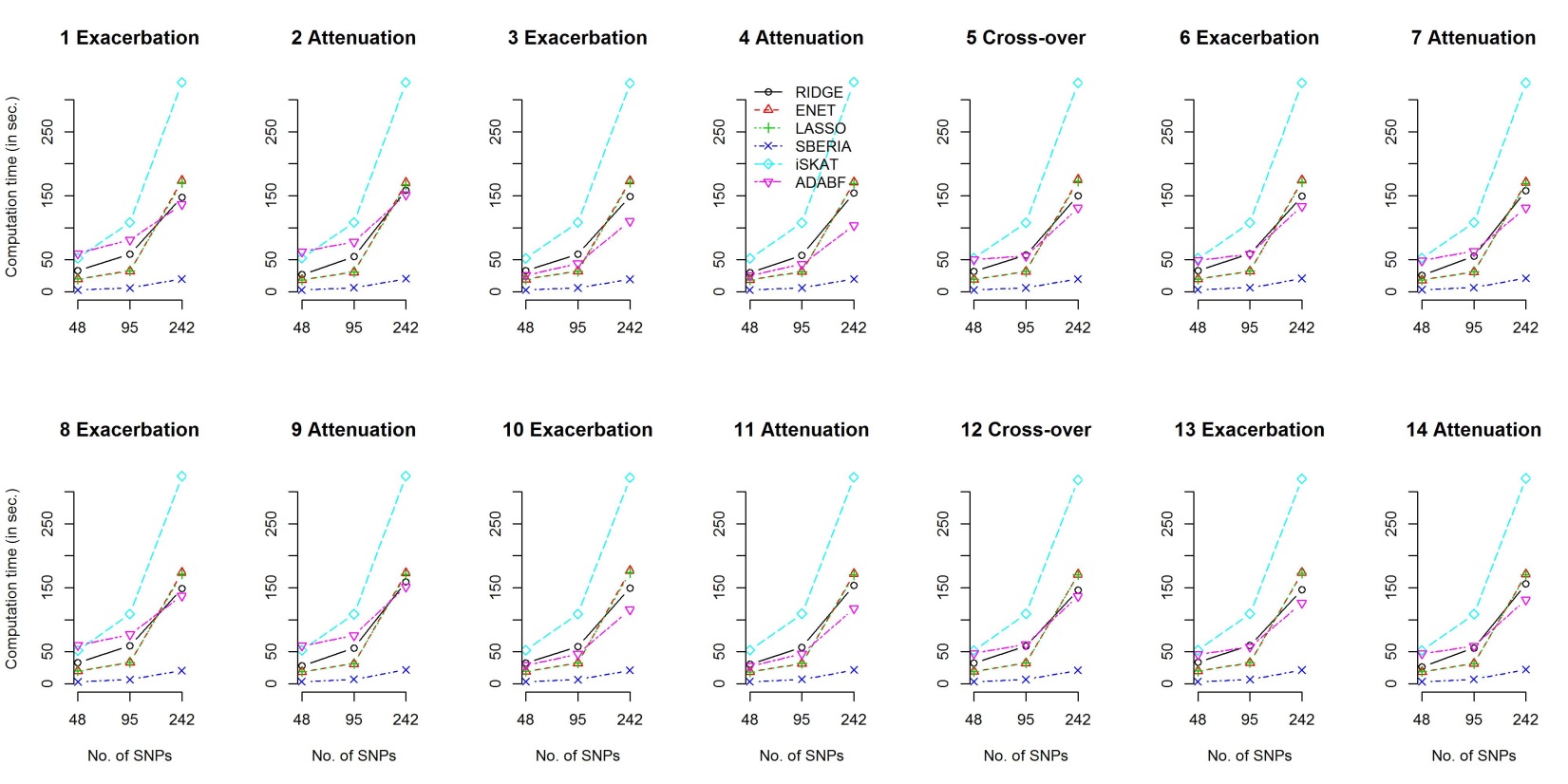


**Figure S29** Average time spent (in seconds) for each simulation replication, under $H_{1}$, for binary traits, $P\left( Y=1 \right)=0.4$, and $P\left( E=1 \right)=0.5$
